# The human MRS2 magnesium-binding domain is a regulatory feedback switch for channel activity

**DOI:** 10.26508/lsa.202201742

**Published:** 2023-02-08

**Authors:** Sukanthathulse Uthayabalan, Neelanjan Vishnu, Muniswamy Madesh, Peter B Stathopulos

**Affiliations:** 1 Department of Physiology and Pharmacology, Schulich School of Medicine and Dentistry, University of Western Ontario , London, Canada; 2 Center for Mitochondrial Medicine, Department of Medicine, University of Texas Health San Antonio, San Antonio, TX, USA

## Abstract

Mitochondrial RNA splicing 2 (MRS2) forms a magnesium (Mg^2+^) entry protein channel in mitochondria. Whereas MRS2 contains two transmembrane domains constituting a pore on the inner mitochondrial membrane, most of the protein resides within the matrix. Yet, the precise structural and functional role of this obtrusive amino terminal domain (NTD) in human MRS2 is unknown. Here, we show that the MRS2 NTD self-associates into a homodimer, contrasting the pentameric assembly of CorA, an orthologous bacterial channel. Mg^2+^ and calcium suppress lower and higher order oligomerization of MRS2 NTD, whereas cobalt has no effect on the NTD but disassembles full-length MRS2. Mutating-pinpointed residues-mediating Mg^2+^ binding to the NTD not only selectively decreases Mg^2+^-binding affinity ∼sevenfold but also abrogates Mg^2+^ binding–induced secondary, tertiary, and quaternary structure changes. Disruption of NTD Mg^2+^ binding strikingly potentiates mitochondrial Mg^2+^ uptake in WT and Mrs2 knockout cells. Our work exposes a mechanism for human MRS2 autoregulation by negative feedback from the NTD and identifies a novel gain of function mutant with broad applicability to future Mg^2+^ signaling research.

## Abstract (precis)

Magnesium (Mg^2+^) binding to the human mitochondrial RNA splicing 2 (MRS2) protein channel amino terminal domain disrupts homomeric interactions and inhibits mitochondrial Mg^2+^ uptake as a negative feedback mechanism.

## Introduction

Magnesium ions (Mg^2+^) are the most abundant divalent cations in eukaryotes, playing universal roles in myriad cell functions (Jahnen-Dechent & Ketteler, 2012). Within mitochondria, Mg^2+^ is an important protein-stabilizing cofactor, forms biologically functional Mg^2+^–ATP complexes, and regulates crucial enzymatic activities. Such roles are achieved through two unique properties of Mg^2+^: (i) the ability to form chelates with important intracellular anionic-ligands (i.e., small molecule or large biomolecule), and (ii) the capability to compete with calcium ions (Ca^2+^) for binding sites on proteins and membranes (Berridge et al, 2000; Moomaw & Maguire, 2008; Chaigne-Delalande et al, 2013). The effects of Mg^2+^ on Ca^2+^-handling proteins significantly influence intracellular Ca^2+^ dynamics and signaling (Gregan et al, 2001; Clapham, 2007; de Baaij et al, 2015).

Total cellular Mg^2+^ concentrations range between ∼17 and 30 mM; however, concentrations of free Mg^2+^ in the cytosol are estimated between ∼0.5 and 1.5 mM (Jung et al, 1990; Rutter et al, 1990; Romani, 2011). Intracellular Mg^2+^ concentrations are strongly buffered and regulated by the combined action of Mg^2+^-binding molecules, Mg^2+^ storage in organelles, and the action of Mg^2+^ channels and exchangers. Remarkably, Mg^2+^ can be mobilized from the ER in response to ligands such as L-lactate, moving into the mitochondria and dramatically modifying metabolism (Daw et al, 2020). Mg^2+^ can also alter the electrophysiological properties of ion channels such as voltage-dependent Ca^2+^ channels and potassium (K^+^) channels and affect the binding affinity of Ca^2+^ to EF-hand–containing proteins (Glancy & Balaban, 2012; Pilchova et al, 2017). All ATP-related biochemical reactions in cells are dependent on Mg^2+^ (Romani, 2007), and extracellular Mg^2+^ also regulates numerous channels such as glutamate receptors and N-methyl-D-aspartate receptors (Kumar, 2015). In addition, this divalent cation contributes to the maintenance of genome stability as a cofactor in DNA repair and protection (Hartwig, 2001).

Unsurprisingly, perturbations in intracellular Mg^2+^ concentrations can cause serious cellular dysfunction. For example, decreases in intracellular free Mg^2+^ lead to defective immune responses (Zhou & Clapham, 2009; Chaigne-Delalande et al, 2013; Kanellopoulou et al, 2019), mutations in the Na^+^/Mg^2+^ exchanger causing chronic intracellular Mg^2+^ deficiency trigger neuronal damage (Kolisek et al, 2013), and overexpression of Mg^2+^ channels is a hallmark of several types of cancer (Trapani & Wolf, 2019), to name a few. More specifically in terms of neuronal disease, the A350V mutation in SLC41A1 enhances Na+-dependent Mg^2+^ efflux by ∼70% in HEK293 cells (Kolisek et al, 2013), and low Mg^2+^ intake in rats and humans leads to loss of dopaminergic neurons (Oyanagi et al, 2006) and increased risk of idiopathic Parkinson’s disease, respectively (Aden et al, 2011). Indeed, decreased free cytosolic Mg^2+^ has been measured in the occipital lobes of Parkinson’s patients (Barbiroli et al, 1999). Transient receptor potential melastatin-6 mutations can also be pathophysiological, resulting in Mg^2+^ malabsorption and renal wasting, where affected individuals show seizures and muscle spasms during infancy (Schlingmann et al, 2002; Schaffers et al, 2018).

Mitochondria have an inner mitochondrial membrane (IMM), separating the mitochondrial matrix (MM) from the intermembrane space, and an outer mitochondrial membrane, enclosing the entire organelle. Unregulated, the highly negative IMM potential (∼−180 mV) (Marchi & Pinton, 2014) would drive catastrophically high concentrations of Mg^2+^ entry into the matrix; nevertheless, the matrix Mg^2+^ concentration is similar to the concentration in the cytoplasm, reinforcing that Mg^2+^ influx into the organelle is tightly controlled to maintain optimal mitochondrial function and bioenergetics (Marchi & Pinton, 2014; Pilchova et al, 2017).

**Figure 1.**
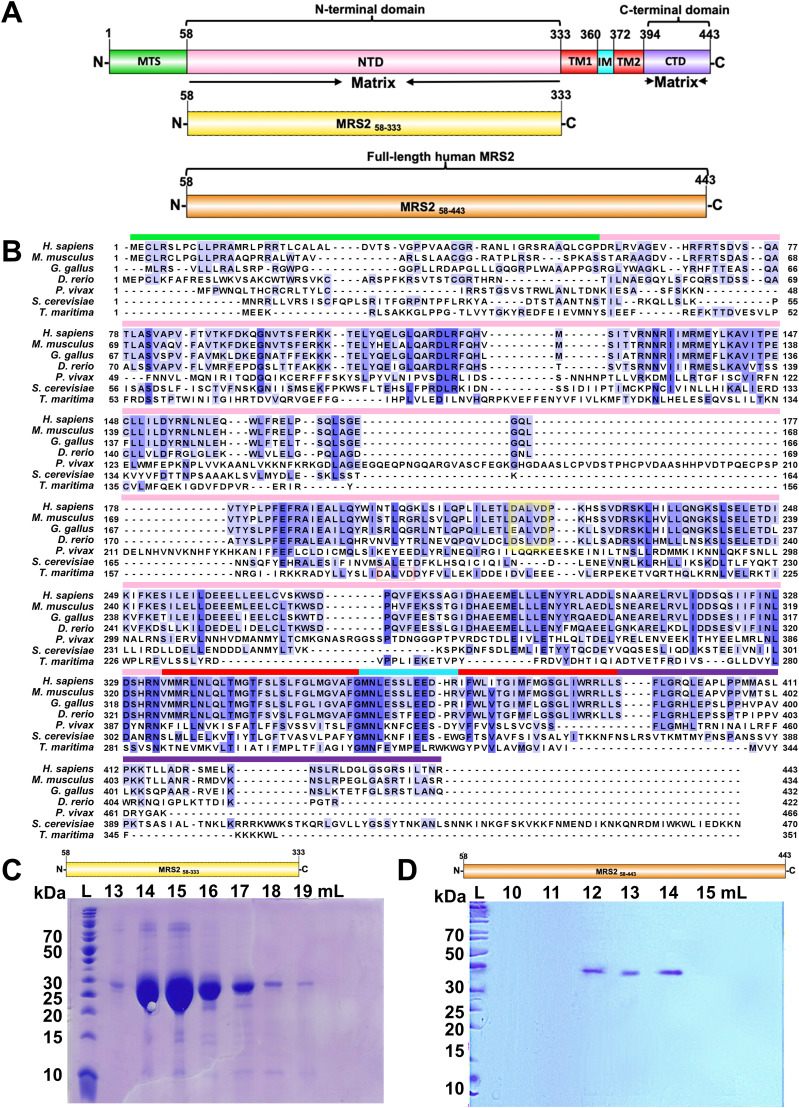
Domain architecture and sequence alignments of MRS2 family proteins. **(A)** Domain architecture of human MRS2. The relative locations of the mitochondrial targeting sequence (MTS, green), amino terminal domain (NTD, magenta), transmembrane 1, 2 (TM1/2, red), intermembrane space (IM, cyan), and C-terminal domain (CTD, violet) are shown. The residue ranges are shown above and below each domain, labeled based on UniProt Q9HD23 and bioinformatics annotations (see the Results section). Below, the N-terminal domain (NTD, yellow) and full-length (orange) constructs engineered in the present study are shown relative to the entire pre-protein. **(B)** Multiple sequence alignment of MRS2 orthologues. Sequences for human (UniProt accession Q9HD23), *Thermotoga maritima* (Q9WZ31), *Saccharomyces cerevisiae* (Q01926), *Plasmodium Vivax* (A0A1G4H438), *Danio rerio* (E7F680), *Gallus gallus* (A0A1D5P665), and *Mus musculus* (Q5NCE8) were aligned using Clustal Omega with defaults (Sievers et al, 2011) and annotated in Jalview (Waterhouse et al, 2009). **(A)** Coloured bars above the human MRS2 sequence mark the boundaries as per the pre-protein in (A). Yellow and red boxes highlight the location of the DALVD sequence in vertebrates and *T*. *maritima*, respectively. Residue numbers are shown at left and right of each entry and blue shades correspond to conserved positions. Note that human MRS2 and bacterial CorA alignment is algorithm-dependent due to poor sequence conservation (see Figs S1 and S2). **(C)** Coomassie blue-stained SDS–PAGE gel showing the elution fractions containing human MRS2_58–333_. **(D)** Coomassie blue–stained SDS–PAGE gel showing the elution fractions containing MRS2_58–443_. In (C, D), elution volumes through an S200 10/300 Gl SEC column are shown at top and ladder “L” bands are shown at left.

Residing within the IMM in mammalian cells, mitochondrial RNA splicing 2 (MRS2) constitutes a major Mg^2+^ entry protein channel into the MM. Deletion of IMM-localized MRS2 abolishes Mg^2+^ influx into the MM, inducing functional defects in mitochondria and promoting cell death (Piskacek et al, 2009; Merolle et al, 2018). MRS2 belongs to the large heterogeneous CorA/Mrs2/Alr1 protein superfamily of Mg^2+^ transporters. This family is characterized by the highly conserved Gly-Met-Asn (GMN) motif at the end of the first transmembrane helix, essential for Mg^2+^ transporter function. Mutations of the GMN motif either completely abolish Mg^2+^ transport or profoundly change the ion selectivity of the channel (Knoop et al, 2005; Palombo et al, 2013; Merolle et al, 2018). Human MRS2 contains a large, amino terminal domain (NTD) oriented within the MM, corresponding to residues 58–333 and consisting of ∼71% of the mature polypeptide chain, two transmembrane (TM1 and TM2) domains connected by a highly conserved intermembrane space loop and a smaller, carboxyl terminal domain also oriented within the MM (Fig 1A).

Although CorA and Mrs2, orthologues of MRS2 in bacteria and yeast, respectively, have been structurally resolved at high resolution (Eshaghi et al, 2006; Lunin et al, 2006; Payandeh & Pai, 2006; Guskov et al, 2012; Pfoh et al, 2012; Khan et al, 2013; Matthies et al, 2016; Johansen et al, 2022), low sequence similarity exists between CorA, Mrs2, and MRS2, especially in the NTDs (Zsurka et al, 2001). Specifically, the sequence similarity between human MRS2 and yeast (*Saccharomyces cerevisiae*) Mrs2 is 55.4% (and only 20.1% through the NTD), whereas the sequence similarity between human MRS2 and bacterial (*Thermotoga maritima*) CorA is 43.3% (and only 17.0% through the NTD) (Fig 1B). To reveal how the prominent MRS2 NTD governs the assembly and function of the full channel, we generated recombinant human NTD protein (MRS2_58–333_; residues 58–333) and full-length human MRS2 (MRS2_58–443_; residues 58–443). Using light scattering and chromatographic approaches, we find that in the absence of divalent cations, the NTD self-associates into a homodimer under dilute conditions, whereas both Mg^2+^ and Ca^2+^, but not cobalt (Co^2+^), suppress the self-association of the domain. In contrast, Co^2+^ disassembles full-length MRS2, whereas Mg^2+^ and Ca^2+^ have no effect on stoichiometry. 8-Anilino-1-naphthalene sulfonate (ANS) and intrinsic fluorescence measurements suggest that Mg^2+^ and Ca^2+^ bind to distinct sites on the NTD with ∼μM and mM affinity, respectively. Importantly, we identify the D216 and D220 as critical residues for Mg^2+^ coordination to the human MRS2 NTD, where mutating these residues decreases Mg^2+^ affinity ∼sevenfold, abrogates Mg^2+^ binding–induced increases in α-helicity and solvent accessible hydrophobicity, and suppresses the Mg^2+^-induced disassembly of the NTD. Finally, using both permeabilized and intact cell models, we show that Mg^2+^ binding to the NTD suppresses the rate of Mg^2+^ uptake into mitochondria as negative feedback. Collectively, our data reveal previously unknown mechanistic insights underlying human MRS2 autoregulation by the large NTD, which has important implications for understanding the crosstalk between MM Mg^2+^ concentrations, bioenergetics, and cell death.

## Results

### MRS2 NTD homodimer assembly is sensitive to divalent cations

**Figure 2.**
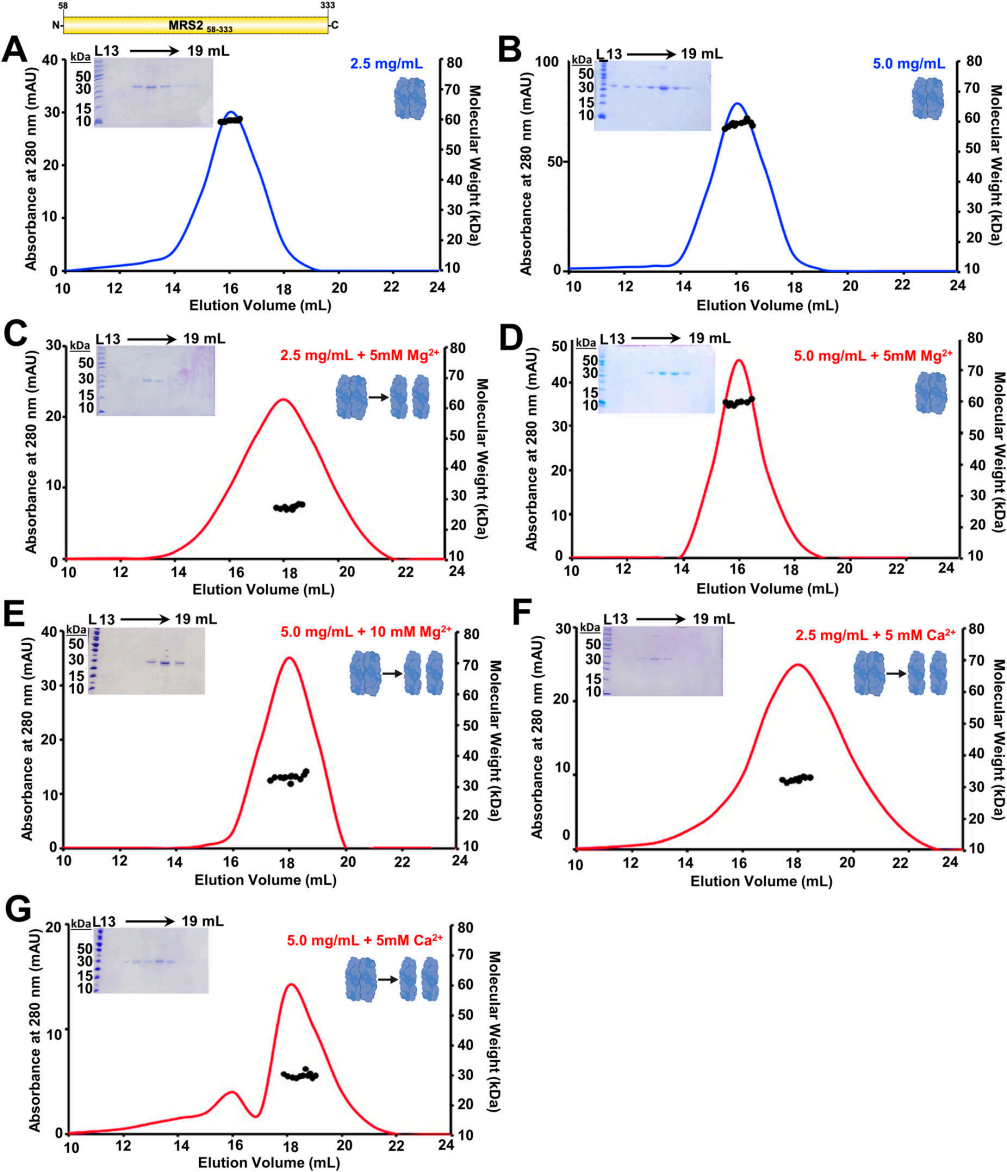
Quaternary structure of MRS2_58–333_ (NTD). **(A, B)** SEC-MALS analysis of MRS2_58–333_ injected at (A) 2.5 mg/ml and (B) 5.0 mg/ml in the absence of divalent cations. **(C, D)** SEC-MALS data of MRS2_58–333_ injected at (C) 2.5 mg/ml and (D) 5.0 mg/ml in the presence of 5 mM MgCl_2_. **(E)** SEC-MALS data of MRS2_58–333_ injected at (E) 5.0 mg/ml MRS2_58–333_ in the presence of 10 mM MgCl_2_. **(F, G)** SEC-MALS data of MRS2_58–333_ injected at (F) 2.5 mg/ml and (G) 5.0 mg/ml in the presence of 5 mM CaCl_2_. In (A, B, C, D, E, F, G), MALS-determined molecular weights are shown through the elution peaks (black circles), left insets show Coomassie blue–stained SDS–PAGE gels of the elution fractions from the 5.0 mg/ml injections and right insets depict the dimerization state of the protein and divalent cation-free and supplemented chromatograms are coloured blue and red, respectively. Elution volumes are indicated at top and ladder (L) molecular weights at left of the gels. Data are representative of n = 3 separate injections from three protein preparations (Table S1) and were acquired using an S200 10/300 Gl column in 20 mM Tris, 150 mM NaCl, and 1 mM DTT, pH 8.0, 10°C.

Given that human *MRS2* encodes only two putative TMs (Fig 1A) and must oligomerize to form a channel pore, we first evaluated the stoichiometry of the MRS2 NTD (i.e., MRS2_58–333_) using size exclusion chromatography with in-line multi-angle light scattering (SEC-MALS). Recombinant MRS2_58–333_ was successfully expressed and isolated with high yield (i.e., ∼8 g/l of culture) and purity (Fig 1C). Although the theoretical monomeric molecular weight of MRS2_58–333_ is ∼32.2 kD, SEC-MALS revealed that, in the absence of divalent cations, MRS2_58–333_ consistently self-associates into a homodimer with estimated molecular weights of 60.9 ± 1.8 kD and 61.3 ± 0.50 kD at 2.5 and 5.0 mg/ml, respectively (Fig 2A and B). Homodimer formation is apparently tight as single elution peaks and no protein concentration dependence on the molecular weight or elution volume in the 0.45–5 mg/ml range was observed (Table S1).

Table S1 Summary of SEC-MALS data for MRS2_58–333_.

SEC-MALS was further used to assess the sensitivity of the homodimer assembly to Mg^2+^ and Ca^2+^ because earlier studies showed divalent cation binding to the CorA NTD regulates channel structure and function (Pfoh et al, 2012; Khan et al, 2013) (see also Discussion section). The presence of 5 mM MgCl_2_ in the elution buffer transitioned the molecular weight of MRS2_58–333_ to monomer at 2.5 mg/ml, with a SEC-MALS molecular weight of 29.4 ± 6.0 kD (Fig 2C). In contrast, MRS2_58–333_ remained dimeric at 5 mg/ml in the presence of 5 mM MgCl_2_, with a molecular weight of 60.06 ± 0.47 kD (Fig 2D). Adding 10 mM MgCl_2_ to the 5 mg/ml sample, however, resulted in a monomeric molecular weight of 32.6 ± 0.5 kD (Fig 2E). Remarkably, adding 5 mM CaCl_2_ to both the 2.5 and 5.0 mg/ml MRS2_58–33_ samples robustly caused monomer formation, with measured molecular weights of 31.4 ± 1.0 and 29.0 ± 0.44 kD, respectively (Fig 2F and G). Supplementation with 5 mM MgCl_2_ to MRS2_58–333_ samples at less than 2.5 mg/ml was sufficient to cause monomerization, and shifts to later elution volumes were consistent with all divalent cation-dependent disassembly observations (Table S1).

### Divalent cations regulate MRS2 assembly in a domain-specific manner

**Figure 3.**
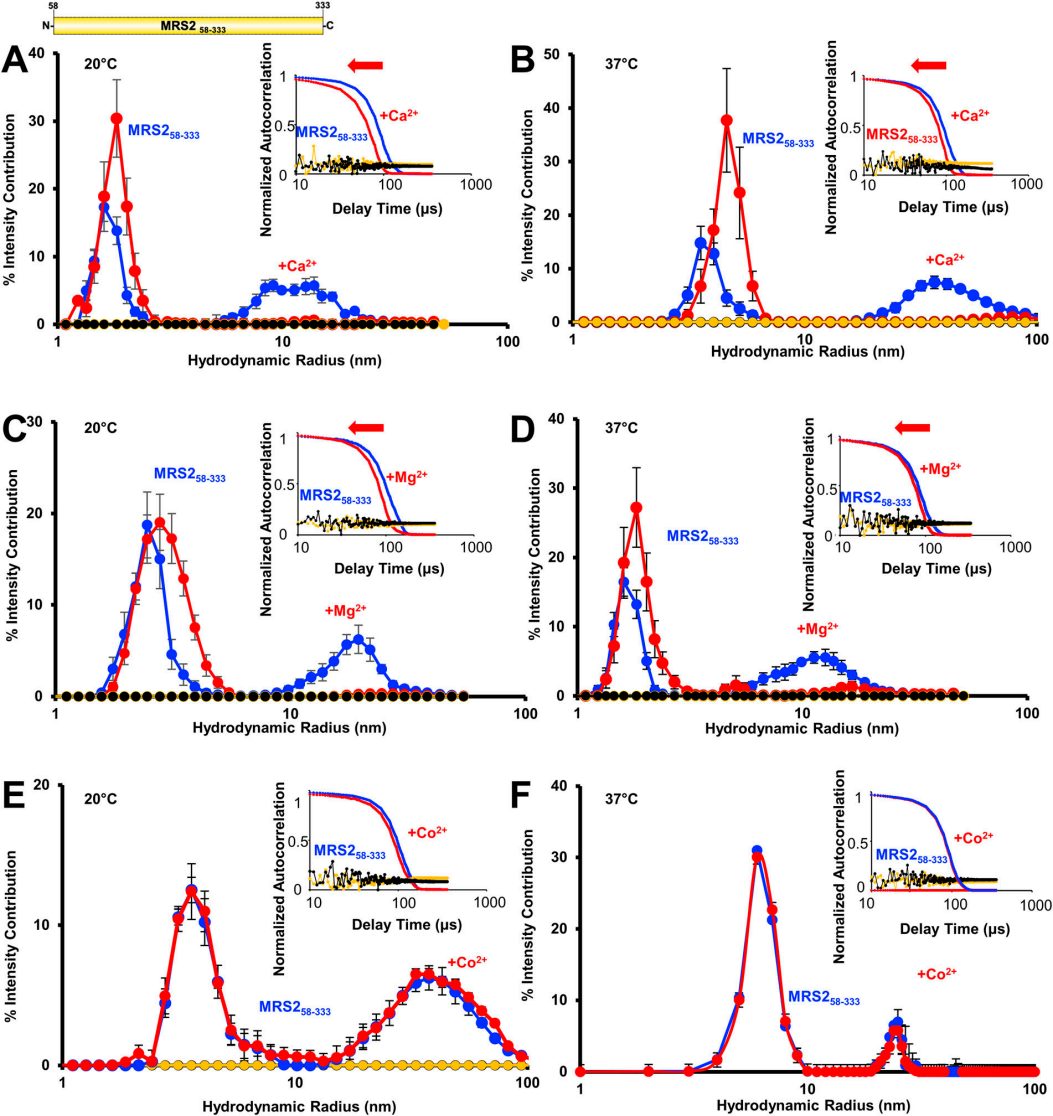
Higher order oligomerization of MRS2_58–333_ (NTD). **(A, B)** Distributions of hydrodynamic radii (R_h_) from the regularization deconvolution of the autocorrelation functions in the presence and absence of 5 mM CaCl_2_ at (A) 20°C and (B) 37°C. **(C, D)** Distributions of R_h_ from the regularization deconvolution of the autocorrelation functions in the presence and absence of 5 mM MgCl_2_ at (C) 20°C and (D) 37°C. **(E, F)** Distributions of R_h_ from the regularization deconvolution of the autocorrelation functions in the presence and absence of 5 mM CoCl_2_ at (E) 20°C and (F) 37°C. In (A, B, C, D, E, F), insets show the divalent cation-induced shifts in the autocorrelation functions, divalent cation-free protein sample data are coloured blue, divalent cation-supplemented protein sample data are red, divalent cation-free buffer control data are black, and divalent cation-supplemented buffer control data are yellow. Inset data are representative, while deconvoluted R_h_ profiles are means ± SEM of n = 3 separate samples from three protein preparations. All data were acquired at 1.25 mg/ml protein in 20 mM Tris, 150 mM NaCl, and 1 mM DTT, pH 8.0.

Because SEC-MALS was performed at 10°C and is accompanied by a large column dilution (i.e., minimum ∼20-fold) that could affect assembly, we used dynamic light scattering (DLS) to assess the distribution of hydrodynamic radii (R_h_) at 1.25 mg/ml in the absence of dilution and at higher temperature (i.e., 20 and 37°C). Bimodal distributions of R_h_ centered at ∼4 and 40 nm were observed for MRS2_58–333_ in the absence of divalent cations at both 20 and 37°C (Fig 3A–F). The addition of either 5 mM CaCl_2_ (Fig 3A and B) or 5 mM MgCl_2_ (Fig 3C and D) at both temperatures eliminated the larger size distributions. The loss of larger R_h_ was supported qualitatively by earlier decays in the autocorrelation functions when compared with divalent cation-free protein samples (Fig 3A–D, insets). The bacterial orthologue of MRS2 and CorA can transport Co^2+^ and Mg^2+^, and Co^2+^ is found in trace levels in mammals (Tapiero et al, 2003; Guskov & Eshaghi, 2012; Czarnek et al, 2015); thus, we also assessed the sensitivity of the MRS2 NTD assembly to Co^2+^. The distribution of R_h_ was not affected by the addition of 5 mM CoCl_2_ at either 20 or 37°C (Fig 3E and F).

**Figure 4.**
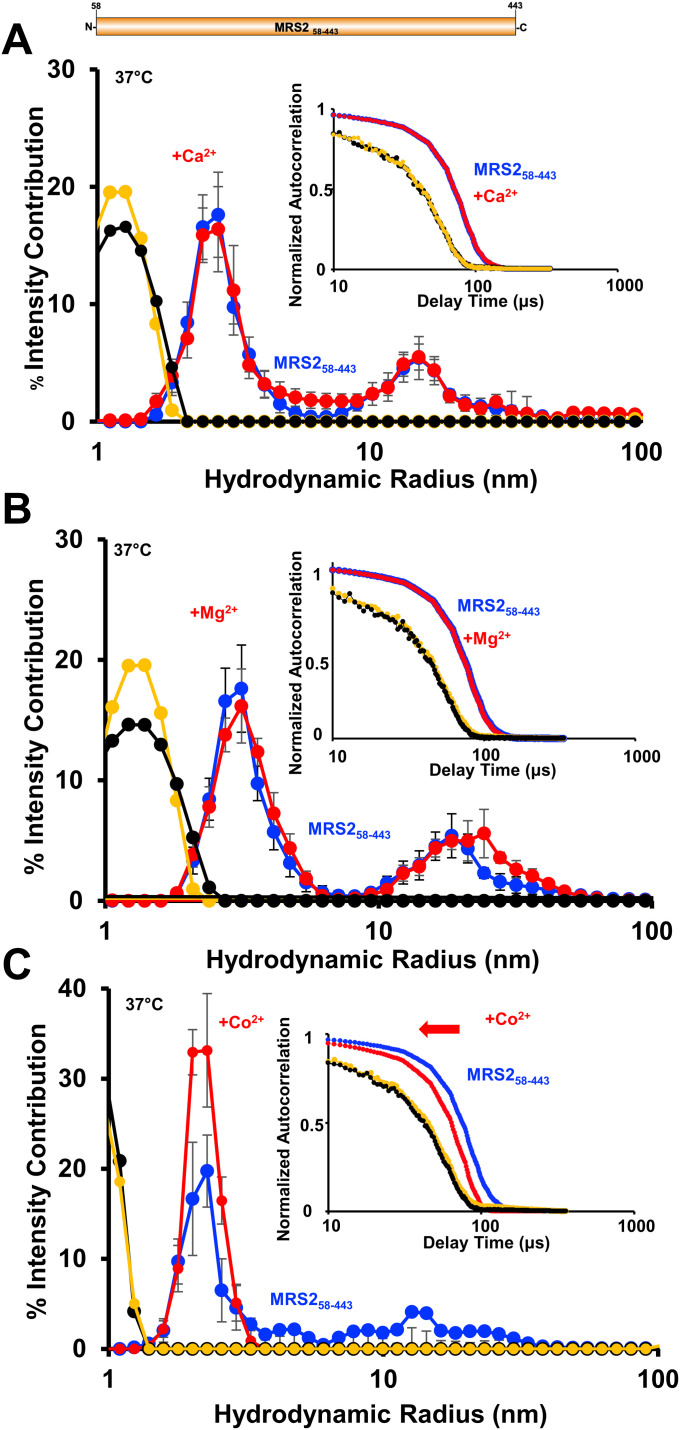
Higher order oligomerization of MRS2_58–443_ (full-length). **(A)** Distributions of R_h_ from the regularization deconvolution of the autocorrelation functions in the presence and absence of 5 mM CaCl_2_. **(B)** Distributions of R_h_ from the regularization deconvolution of the autocorrelation functions in the presence and absence of 5 mM MgCl_2_. **(C)** Distributions of R_h_ from the regularization deconvolution of the autocorrelation functions in the presence and absence of 5 mM CoCl_2_. In (A, B, C), insets show the divalent cation-induced shifts in the autocorrelation functions, divalent cation-free protein sample data are coloured blue, divalent cation-supplemented protein sample data are red, divalent cation-free buffer control data are black and divalent cation-supplemented buffer control data are yellow. Inset data are representative, whereas deconvoluted R_h_ profiles are means ± SEM of n = 3 separate samples from three protein preparations. All data were acquired at 0.5 mg/ml in 20 mM Tris, 150 mM NaCl, 1 mM DTT, and 10 mM CHAPS, pH 8.0, 37°C.

Next, we evaluated the effects of Mg^2+^, Ca^2+^, and Co^2+^ on the assembly of the full-length protein. Full-length MRS2, excluding the mitochondrial targeting sequence, (MRS2_58–443_), was successfully expressed and isolated with high purity (Fig 1D). The experimental buffer for MRS2_58–443_ included CHAPS and showed autocorrelation functions consistent with the presence of ∼1–1.5 nm micelles at 37°C (Fig 4A–C). MRS2_58–443_ samples showed autocorrelation functions with later decay times compared with buffer alone, which were deconvoluted to R_h_ distributions centered at ∼4 and ∼20 nm at 37°C (Fig 4A–C). In contrast to the NTD data, changes in autocorrelation functions and size distributions were not observed with MRS2_58–443_ when supplemented with either 5 mM MgCl_2_ or 5 mM CaCl_2_ (Fig 4A and B). Remarkably, 5 mM CoCl_2_ completely abrogated the larger R_h_ distributions, which was qualitatively supported by autocorrelation function shifts to earlier decay times (Fig 4C).

Applying a cumulative deconvolution to extract one weight-averaged R_h_ from all autocorrelation functions reinforced the regularization/polydisperse deconvolution trends described above. Specifically, CoCl_2_ caused a robust decrease in the weight-averaged R_h_ for full-length MRS2, but not the NTD; MgCl_2_ and CaCl_2_ decreased R_h_ for the NTD, but not full-length MRS2 (Table S2). Collectively, these data suggest a domain-specific sensitivity to divalent cations, where NTD disassembly is promoted by Mg^2+^ and Ca^2+^, whereas Co^2+^ de-oligomerizes full-length MRS2 because of sensitivity outside the NTD.

Table S2 Summary of cumulants fits for MRS2_58–443_ and MRS2_58–333_.

### Mg^2+^ and Ca^2+^ bind to distinct sites on the MRS2 NTD with disparate affinities

**Figure 5.**
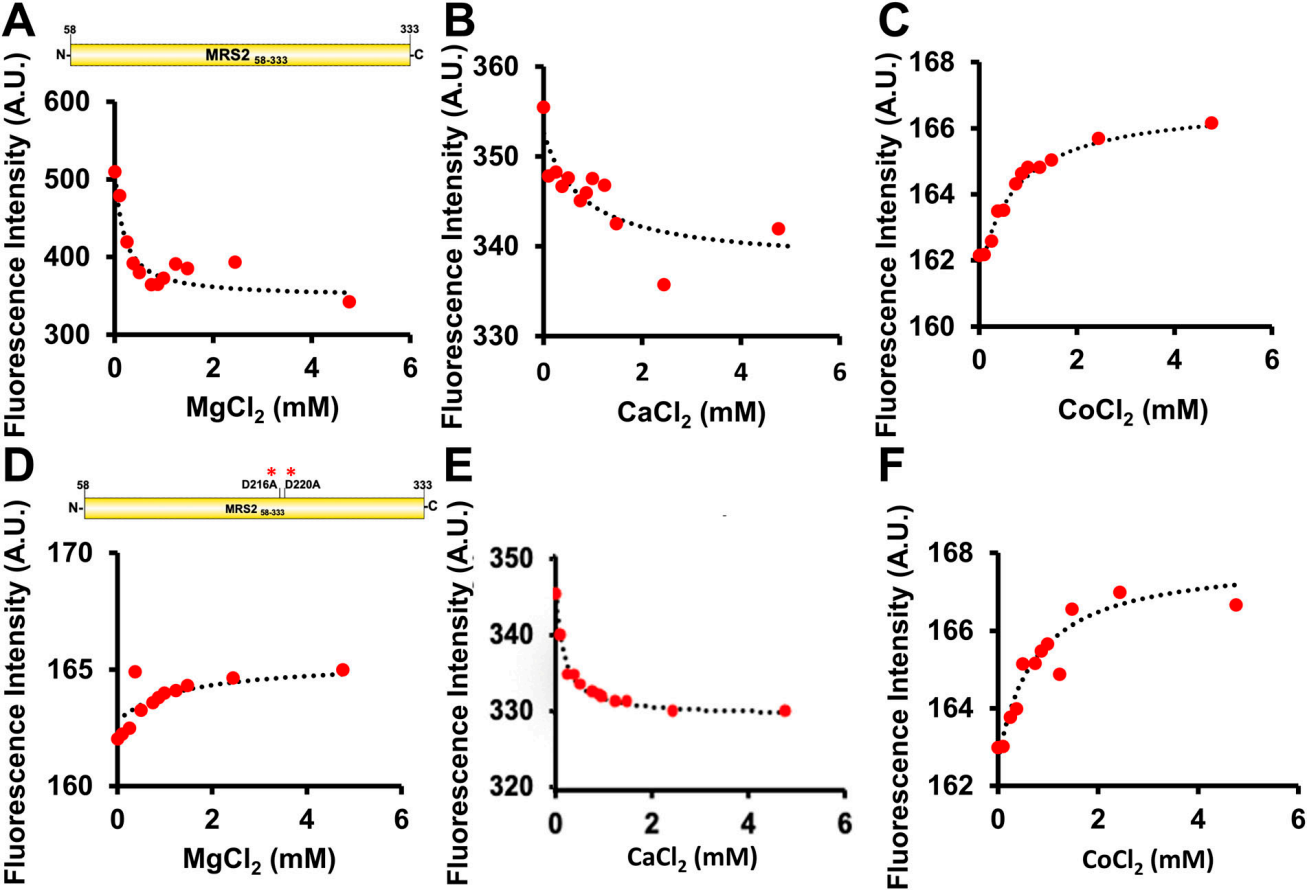
Divalent cation binding affinity to MRS2_58–333_ and MRS2_58–333_ D216A/D220A (NTD). **(A)** Changes in intrinsic fluorescence emission intensity of MRS2_58–333_ as a function of increasing MgCl_2_ concentration. **(B)** Changes in intrinsic fluorescence emission intensity of MRS2_58–333_ as a function of increasing CaCl_2_ concentration. **(C)** Changes in intrinsic fluorescence emission intensity of MRS2_58–333_ as a function of increasing CoCl_2_ concentration. **(D)** Changes in intrinsic fluorescence emission intensity of MRS2_58–333_ D216A/D220A as a function of increasing MgCl_2_ concentration. **(E)** Changes in intrinsic fluorescence emission intensity of MRS2_58–333_ D216A/D220A as a function of increasing CaCl_2_ concentration. **(F)** Changes in intrinsic fluorescence emission intensity of MRS2_58–333_ D216A/D220A as a function of increasing CoCl_2_ concentration. In (A, B, C, D, E, F), data (red circles) show intrinsic fluorescence intensities at 330 nm as a function of increasing divalent cation concentration and are representative of n = 3 separate experiments (Table S3) performed from three protein preparations. The dashed lines through the data fit to a one-site binding model that accounts for protein concentration. All experiments were performed with 0.1 µM protein in 20 mM Tris, 150 mM NaCl, and 1 mM DTT, pH 8.0, at 22.5°C.

Given that both Mg^2+^ and Ca^2+^ dissociate MRS2_58–333_, which contains 3×Trp and 7×Tyr residues, we next used changes in intrinsic fluorescence to evaluate divalent cation binding. Fluorescence emission spectra were acquired using an excitation wavelength of 280 nm as a function of increasing MgCl_2_, CaCl_2_, and CoCl_2_ concentrations. The intensities of the fluorescence emission spectra decreased as a function of increasing MgCl_2_ (Fig 5A) and CaCl_2_ (Fig 5B) concentrations. Both MgCl_2_ and CaCl_2_ effects were saturable; however, the intensity decreased by ∼32% with Mg^2+^ and only ∼5% with Ca^2+^, suggesting distinct structural effects and/or binding sites. In contrast, titration with CoCl_2_ caused small increases of ∼2% in fluorescence (Fig 5C). Fitting the binding curves to a one-site binding model that accounts for protein concentrations revealed apparent equilibrium dissociation constants (K_d_)s of ∼0.14 ± 0.03, 1.01 ± 0.26, and 0.68 ± 0.30 mM for Mg^2+^, Ca^2+^, and Co^2+^ interactions, respectively (Table S3).

Table S3 Summary of the fitted equilibrium dissociation constants (K_d_).

**Figure S1.**
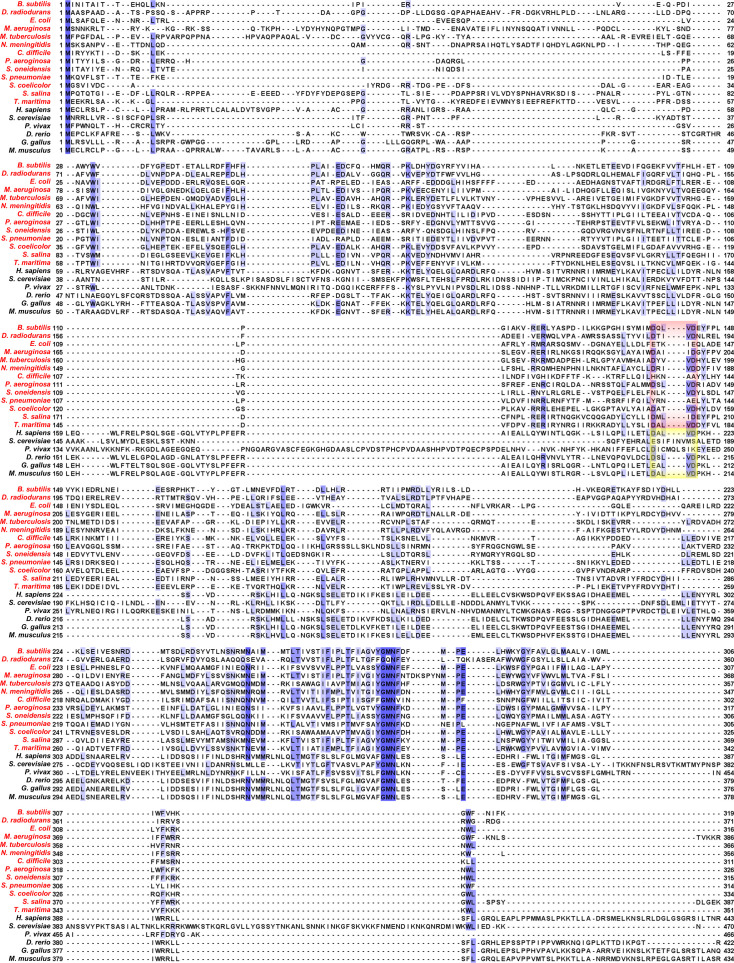
T-Coffee–mediated multiple sequence alignment of MRS2 orthologues enriched with bacterial CorA sequences. The MRS2/CorA sequences are *Bacillus subtilis* (NCBI NP_388681.1), *Deinococcus radiodurans* (NCBI WP_162177618.1), *Escherichia coli* (NCBI AAA67612.1), *Microcystis auruginosa* (NCBI WP_061430759.1), *Mycobacterium tuberculosis* (NCBI CCE36759.1), *Neisseria meningitidis* (NCBI SPY01457.1), *Clostridioides difficile* (NCBI AJP11861.1), *Pseudomonas aeruginosa* (NCBI NP_253955.1), *Shewanella oneidensis* (NCBI AAN54992.1), *Streptococcus pneumoniae* (NCBI VDG79179.1), *Streptomyces coelicolor* (NCBI QFI41861.1), *Synechocystis salina* (NCBI WP_194018573.1), *Thermotoga maritima* (UniProt Q9WZ31), human (UniProt Q9HD23), *Saccharomyces cerevisiae* (UniProt Q01926), *Plasmodium Vivax* (UniProt A0A1G4H438), *Danio rerio* (UniProt E7F680), *Gallus gallus* (UniProt A0A1D5P665), and *Mus musculus* (UniProt Q5NCE8). The 13 bacterial species included (red) are the complete list curated in the NCBI Landmark database spanning a diverse, nonredundant, and wide taxonomic range. The alignment was performed using default settings in T-Coffee (Notredame et al, 2000) and annotated using Jalview (Waterhouse et al, 2009), where residue numbers are indicated at left and right of each line entry and blue shades correspond to conserved positions. The red and yellow boxes highlight the location of the DALVD sequences in the bacteria and higher organisms, respectively.

**Figure S2.**
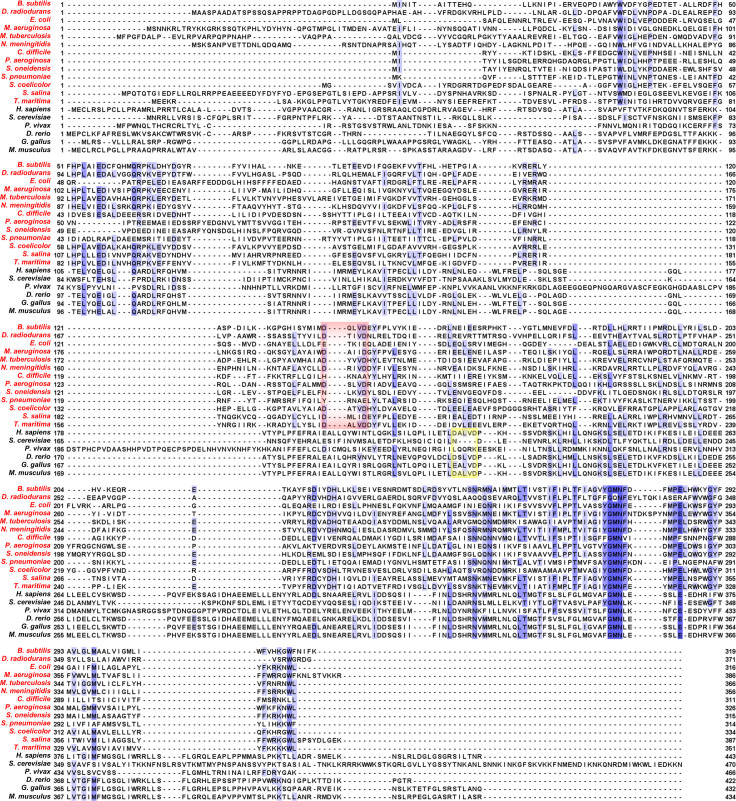
Clustal Omega-mediated multiple sequence alignment of MRS2 orthologues enriched with bacterial CorA sequences. The MRS2/CorA sequences are *Bacillus subtilis* (NCBI NP_388681.1), *Deinococcus radiodurans* (NCBI WP_162177618.1), *Escherichia coli* (NCBI AAA67612.1), *Microcystis aeruginosa* (NCBI WP_061430759.1), *Mycobacterium tuberculosis* (NCBI CCE36759.1), *Neisseria meningitidis* (NCBI SPY01457.1), *Clostridioides difficile* (NCBI AJP11861.1), *Pseudomonas aeruginosa* (NCBI NP_253955.1), *Shewanella oneidensis* (NCBI AAN54992.1), *Streptococcus pneumoniae* (NCBI VDG79179.1), *Streptomyces coelicolor* (NCBI QFI41861.1), *Synechocystis salina* (NCBI WP_194018573.1), *Thermotoga maritima* (UniProt Q9WZ31), human (UniProt Q9HD23), *Saccharomyces cerevisiae* (UniProt Q01926), *Plasmodium vivax* (UniProt A0A1G4H438), *Danio rerio* (UniProt E7F680), *Gallus gallus* (UniProt A0A1D5P665), and *Mus musculus* (UniProt Q5NCE8). The 13 bacterial species included (red) are the complete list curated in the NCBI Landmark database spanning a diverse, nonredundant, and wide taxonomic range. The alignment was performed using default settings in Clustal Omega (Sievers et al, 2011) and annotated using Jalview (Waterhouse et al, 2009), where residue numbers are indicated at left and right of each line entry and blue shades correspond to conserved positions. The red and yellow boxes highlight the location of the DALVD sequences in the bacteria and higher organisms, respectively.

We next attempted to pinpoint the residues involved in Mg^2+^ coordination using the CorA crystal structure (4EED.pdb) (Pfoh et al, 2012) as a guide. Note that available yeast Mrs2 structures do not resolve any Mg^2+^ ions bound to the NTD. The *T*. *maritima* CorA crystal structure shows that two Asp residues, separated by three residues (i.e., DALVD) are involved in Mg^2+^ coordination at one site. Remarkably, human MRS2 contains the same DALVD sequence stretch in the homologous domain, which we posited could similarly coordinate Mg^2+^ (Fig 1B). However, sequence-based alignment of the bacterial and vertebrate DALVD regions are algorithm-dependent because of the poor sequence conservation: T-Coffee (Notredame et al, 2000) aligns the human and *T*. *maritima* DALVD stretches as conserved (Fig S1), whereas Clustal Ω (Sievers et al, 2011) does not (Figs 1B and S2). Moreover, the AlphaFold2 prediction for human MRS2 (see the Discussion section) agrees with the non-conserved Clustal Ω positioning of bacterial and vertebrate DALVD regions. Thus, our use of the term DALVD with respect to human MRS2 refers to the stretch of polypeptide chain containing the D216 and D220 residues and does not imply a motif that is conserved with *T*. *maritima* CorA DALVD. We use the term DALVD to highlight that the two Asp residues are at positions *i* and *i*+4 of this sequence stretch with no intervening helix-breaking residues, putatively orienting these side chains on the same side of an α-helix and adjacent in three-dimensional (3D) space and permitting both Asp to interact with the same Mg^2+^ ion.

After creating a D216A/D220A MRS2_58–333_ double mutant, we reassessed divalent cation binding by intrinsic fluorescence. A double mutant was created because both Asp side chains coordinate the same Mg^2+^ ion in the CorA DALVD sequence, both Asp side chains are close in 3D space in the α-helix where most of the human MRS2 region is predicted to exist by AlphaFold2 (see the Discussion section), and the sub-mM Mg^2+^ K_d_ (Table S3) suggests both Asp are involved in the coordination. Not only did the D216A/D220A mutant show a small increase in fluorescence (Mg^2+^ causes a large decrease in the fluorescence intensity of WT MRS2_58–333_; see above) but also a markedly suppressed intensity change as a function of increasing MgCl_2_, consistent with perturbation of Mg^2+^ binding (Fig 5D). In contrast, the CaCl_2_ and CoCl_2_ effects were similar to data acquired using WT MRS2_58–333_ (Fig 5E and F). Indeed, fitting the datasets to one-site binding models revealed apparent equilibrium dissociation constants (K_d_) of ∼0.98 ± 0.25, 0.74 ± 0.49, and 1.37 ± 0.51 mM (Table S3), consistent with disruption of the Mg^2+^ interactions but not Ca^2+^ or Co^2+^.

Taken together, these data suggest that Mg^2+^ and Ca^2+^ bind to distinct sites on the MRS2NTD, with D216 and D220 mediating interactions with Mg^2+^.

### Mg^2+^ enhances whereas Ca^2+^ suppresses solvent exposed hydrophobicity of MRS2 NTD

**Figure S3.**
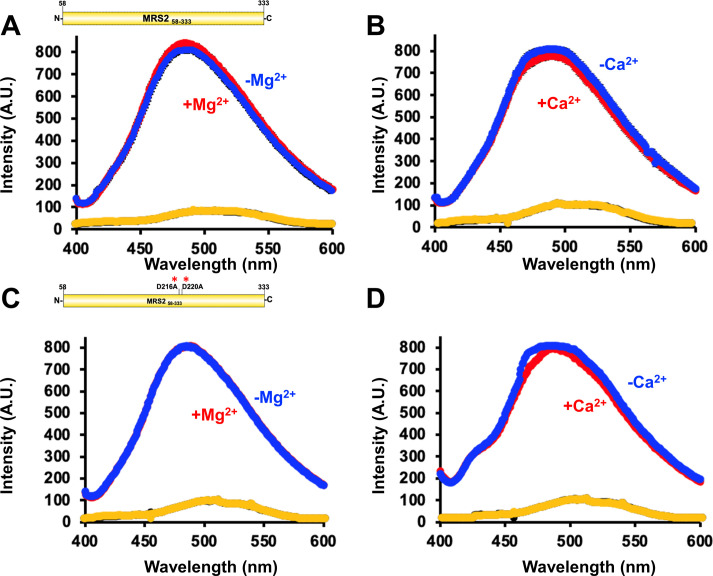
Complete ANS fluorescence emission spectra of MRS2_58–333_ and MRS2_58–333_ D216A/D220A (NTD). **(A)** ANS emission spectra of MRS2_58–333_ in the absence (blue) and presence (red) of 5 mM MgCl2. **(B)** ANS emission spectra of MRS2_58–333_ in the absence (blue) and presence (red) of 5 mM CaCl2. **(C)** ANS emission spectra of MRS2_58–333_ D216A/D220A in the absence (blue) and presence (red) of 5 mM MgCl_2_. **(D)** ANS emission spectra of MRS2_58–333_ D216A/D220A in the absence (blue) and presence (red) of 5 mM CaCl2. In (A, B, C, D), buffer only spectra acquired in the absence and presence divalent cation are coloured black and yellow, respectively. Data are means ± SEM of n = 3 separate experiments from three protein preparations. Data were acquired using 30 µM protein and 50 µM ANS in 20 mM Tris, 150 mM NaCl, and 1 mM DTT, pH 8.0, at 15°C.

**Figure 6.**
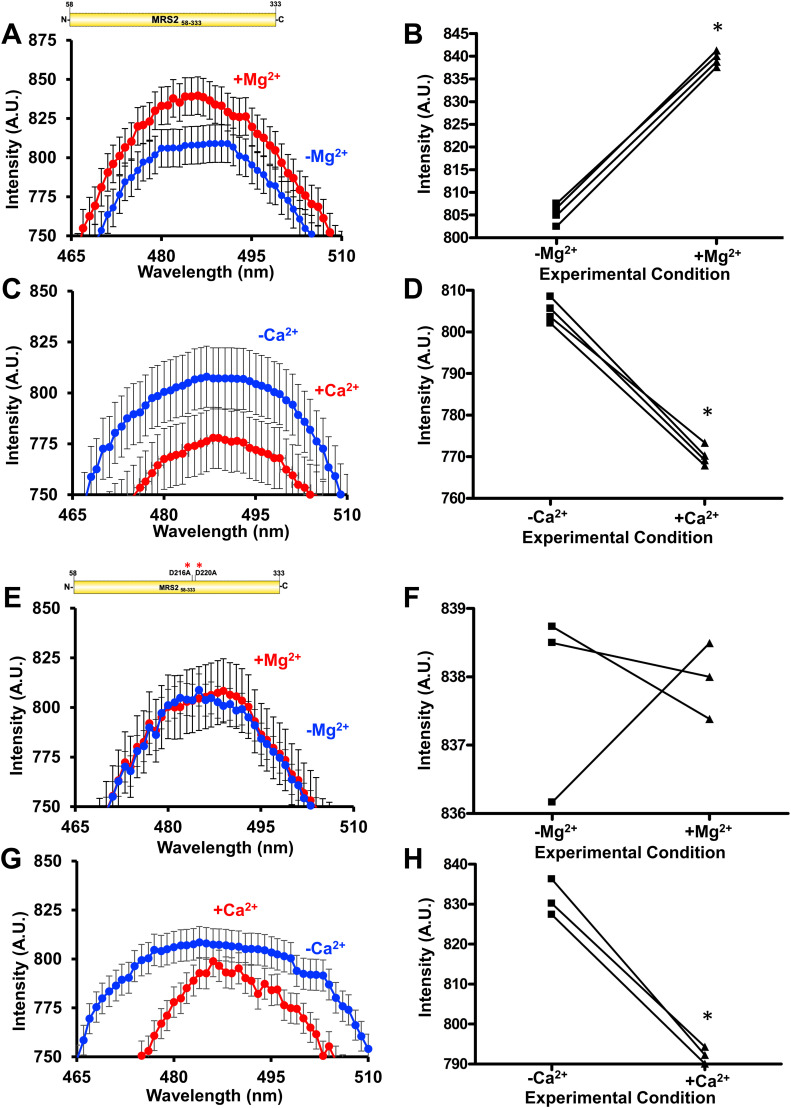
Solvent-exposed hydrophobicity of MRS2_58–333_ and MRS2_58–333_ D216A/D220A (NTD). **(A, B)** ANS fluorescence emission spectra of (A) MRS2_58–333_ in the absence (blue) and presence (red) of 5 mM MgCl_2_ and (B) paired statistical comparisons of the peak intensities. **(C, D)** ANS fluorescence emission spectra of (C) MRS2_58–333_ in the absence (blue) and presence (red) of 5 mM CaCl_2_ and (D) paired statistical comparisons of the peak intensities. **(E, F)** ANS fluorescence emission spectra of (E) MRS2_58–333_ D216A/D220A in the absence (blue) and presence (red) of 5 mM MgCl_2_ and (F) paired statistical comparisons of the peak intensities. **(G, H)** ANS fluorescence emission spectra of (G) MRS2_58–333_ D216A/D220A in the absence (blue) and presence (red) of 5 mM CaCl_2_ and (H) paired statistical comparisons of the peak intensities. In (A, C, E, G), data are means ± SEM of n = 3 separate samples from three protein preparations. In (B, D, F, H), comparisons are paired *t* test analyses, where *P < 0.05. ANS binding experiments were performed using 30 µM protein and 50 µM ANS in 20 mM Tris, 150 mM NaCl, and 1 mM DTT, pH 8.0, at 15°C.

Given the changes in stoichiometry observed by DLS and SEC-MALS, we next assessed the solvent-exposed hydrophobicity of MRS2_58–333_ in the absence and presence of Mg^2+^ and Ca^2+^ by monitoring extrinsic ANS fluorescence. ANS binds to solvent accessible hydrophobic regions on biomolecules, resulting in a blue-shifted fluorescence emission maximum and increased intensity (Stryer, 1965). Baseline fluorescence emission spectra of ANS in the presence of buffer alone were insensitive to the addition of 5 mM MgCl_2_ or 5 mM CaCl_2_ (Fig S3A and B). Indeed, ANS binding was detected in the presence of 2.5 mg/ml MRS2_58–333_, as evidenced by the blue-shifted fluorescence emission maximum and increased intensity compared with the buffer controls (Fig S3A and B). Supplementing the protein samples with 5 mM MgCl_2_ caused a small but significant increase in ANS fluorescence intensity, suggesting enhanced exposed hydrophobicity (Fig 6A and B). Conversely, supplementation with 5 mM CaCl_2_ caused a small but significant decrease in ANS fluorescence intensity, indicating decreased solvent exposed hydrophobicity (Fig 6C and D).

We next performed a similar set of experiments with the D216A/D220A MRS2_58–333_ protein. Consistent with our observation that this double mutant disrupts Mg^2+^ but not Ca^2+^ binding to the NTD; ANS emission spectra in the presence of protein showed no differences with or without MgCl_2_ supplementation (Figs 6E and F and S3C), whereas CaCl_2_ supplementation caused a small but significant decrease in ANS fluorescence intensity (Figs 6G and H and S3D). This ANS binding data reinforces the notion of disparate Ca^2+^- and Mg^2+^-binding sites and suggests that these divalent cations may cause distinct MRS2 NTD conformational changes.

### D216A/D220A mitigates Mg^2+^-dependent disassembly of the MRS2 NTD

**Figure 7.**
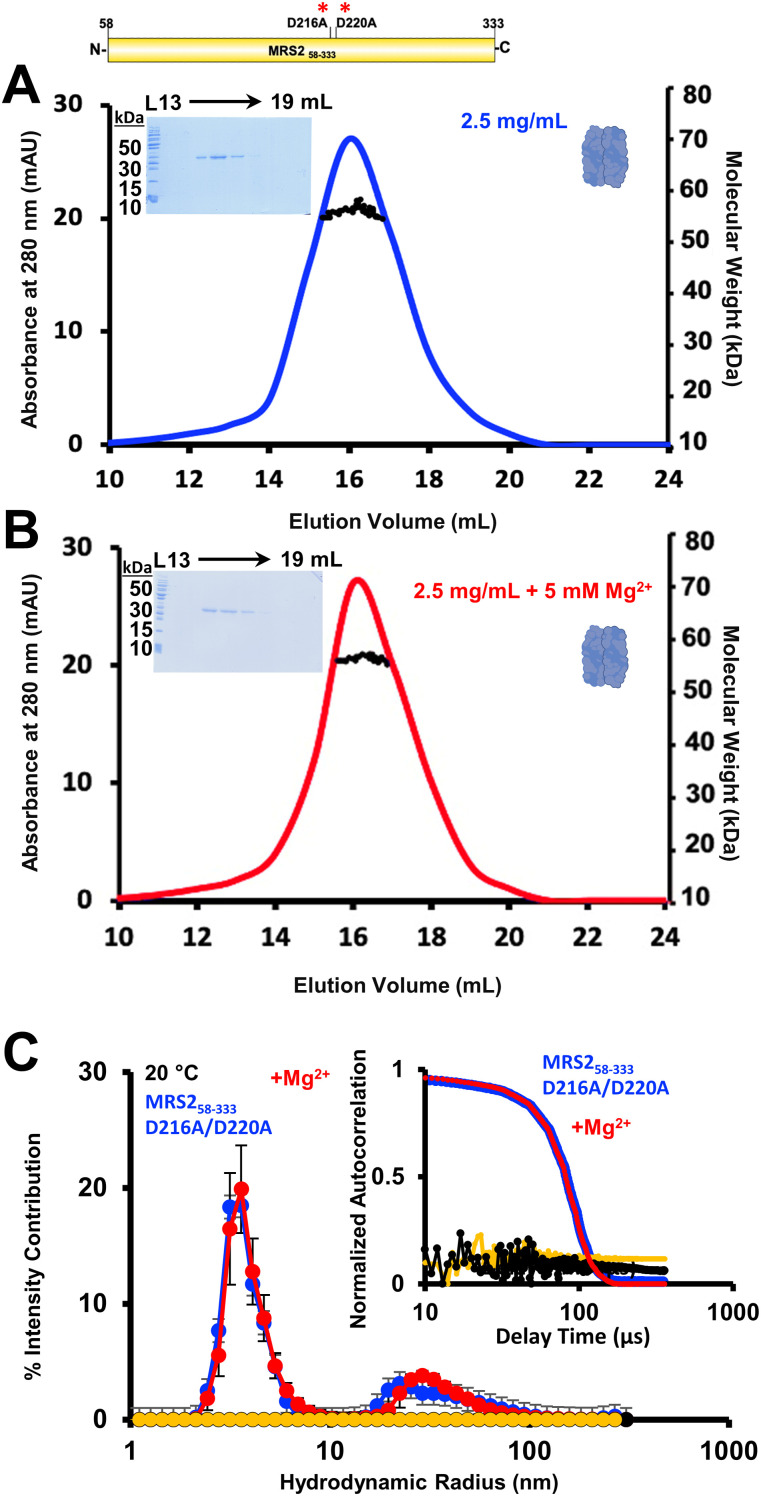
Quaternary structure and higher order oligomerization of MRS2_58–333_ D216A/D220A (NTD). **(A, B)** SEC-MALS analysis of MRS2_58–333_ D216A/D220A injected at 2.5 mg/ml in the (A) absence or (B) presence of 5 mM MgCl_2_. **(C)** DLS analysis of MRS2_58–333_ D216A/D220A at 1.25 mg/ml. The distributions of R_h_ from the regularization deconvolution of the autocorrelation functions are shown in the presence and absence of 5 mM MgCl_2_ at 20°C. In (A, B), MALS-determined molecular weights are shown through the elution peaks (black circles), left insets show Coomassie blue–stained SDS–PAGE gels of the elution fractions from the 2.5 mg/ml injections and right insets depict the dimerization state of the protein. Elution volumes are indicated at top and ladder “L” molecular weights at left of the gels. Data are representative of n = 3 separate injections from three protein preparations (Table S1) and were acquired using an S200 10/300 Gl column in 20 mM Tris, 150 mM NaCl, and 1 mM DTT, pH 8.0, 10°C. In (C), inset show the Mg^2+^-induced shift in the autocorrelation functions, Mg^2+^-free protein sample data are coloured blue, Mg^2+^-supplemented protein sample data are red, Mg^2+^-free buffer control data are black, and Mg^2+^-supplemented buffer control data are yellow. Inset data are representative, while deconvoluted R_h_ profiles are means ± SEM of n = 3 separate samples from three protein preparations. Data were acquired in 20 mM Tris, 150 mM NaCl, and 1 mM DTT, pH 8.0, 20°C.

Next, we tested whether the D216A/D220A double mutation could abolish the Mg^2+^-dependent monomerization and decreased R_h_ observed with WT MRS2 NTD. In the absence of the divalent cation, SEC-MALS revealed that D216A/D220A MRS2_58–333_ elutes as a homodimer with a molecular weight of 59.7 ± 2.0 kD when injected at 2.5 mg/ml (Fig 7A), similar to WT MRS2_58–333_ (Table S1). In contrast to WT evaluated at 2.5 mg/ml, however, the SEC-MALS–determined molecular weight of the double mutant remained dimeric (i.e., 59.0 ± 2.4 kD) after the addition of 5 mM MgCl_2_ (Fig 7B). We also assessed whether Mg^2+^ could alter R_h_ of the D216A/D220A MRS2_58–333_ by DLS. Addition of 5 mM MgCl_2_ neither altered the distribution of R_h_ nor the autocorrelation function compared with samples evaluated in the absence of the cation (Fig 7C). It is to be noted that a bimodal distribution of R_h_ centered at ∼4 and ∼40 nm was observed with the D216A/D220A MRS2_58–333_ protein (Fig 7C), similar to WT.

Together, these light scattering analyses demonstrate that Mg^2+^-dependent disassembly of the MRS2 NTD requires the D216 and D220 residues, where double mutation to Ala abrogates quaternary structure sensitivity to the cation.

### D216A/D220A abrogates increased α-helicity and thermal stability in the MRS2 NTD caused by Mg^2+^ binding

**Figure 8.**
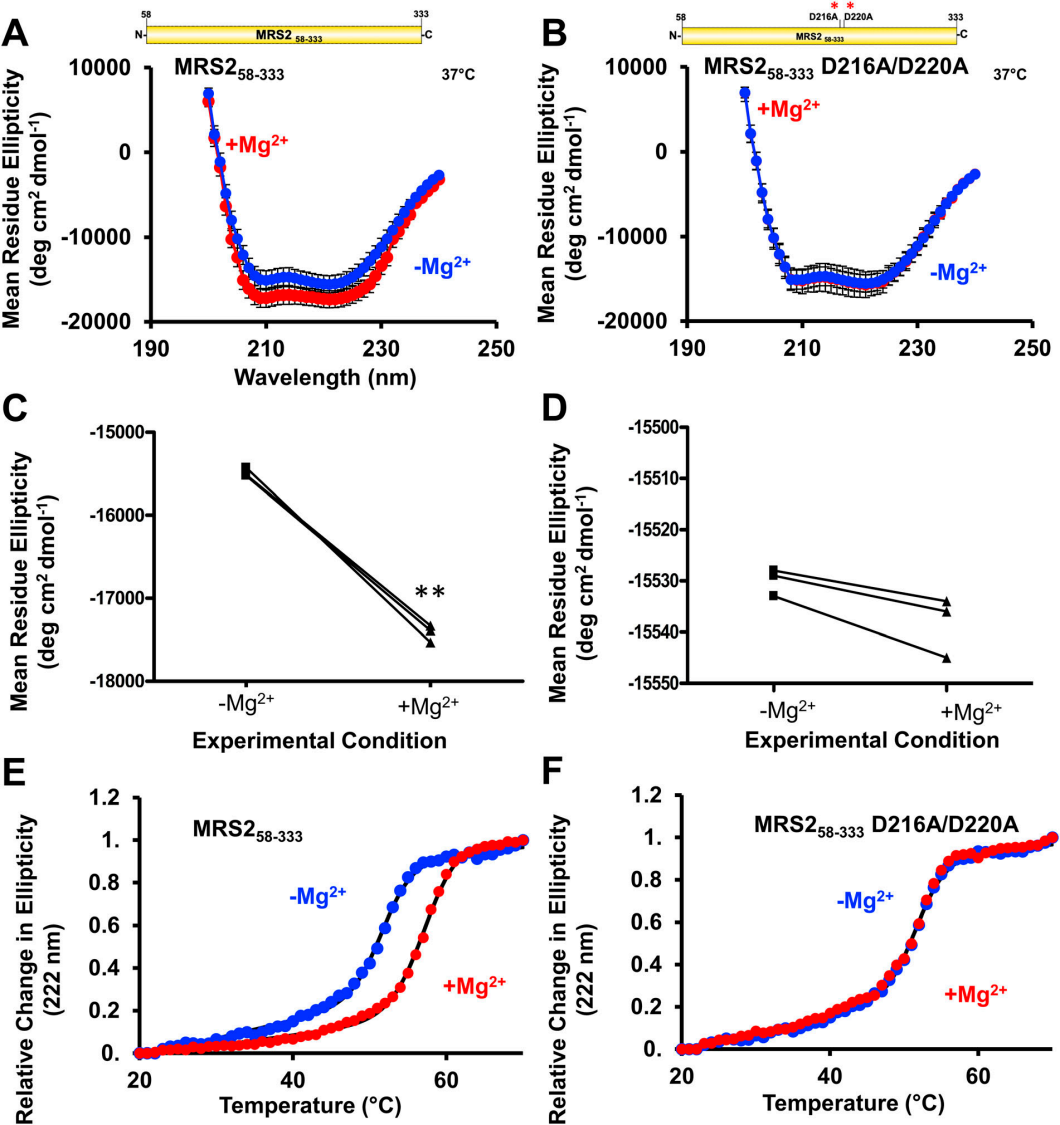
Secondary structure and thermal stability of MRS2_58–333_ and MRS2_58–333_ D216A/D220A (NTD). **(A, B)** Far-UV CD spectra of (A) MRS2_58–333_ and (B) MRS2_58–333_ D216A/D220A in the absence (blue) and presence (red) of 5 mM MgCl_2_. **(C, D)** Statistical comparisons of Mg^2+^-induced changes in mean residue ellipticity at 222 nm for (C) MRS2_58–333_ and (D) MRS2_58–333_ D216A/D220A. **(E, F)** Changes in mean residue ellipticity (222 nm) as a function of increasing temperature (i.e., thermal stability) in the absence (blue) and presence (red) of 5 mM MgCl_2_ for € MRS2_58–333_ and (F) MRS2_58–333_ D216A/D220A. In (A, B), data are means ± SEM of n = 3 experiments with samples from three protein preparations. In (A, B, C, D), comparisons are paired *t* test analyses of data from (A, B), where **P < 0.01. In (E, F), data (circles) are representative of n = 3 separate experiments with samples from three protein preparations, and solid black lines are Boltzmann sigmoidal fits through the data to extract apparent midpoints of temperature denaturation (T_m_). All far-UV CD experiments were acquired with 0.5 mg/ml protein in 20 mM Tris, 150 mM NaCl, and 1 mM DTT, pH 8.0, with spectra acquired at 37°C.

**Figure S4.**
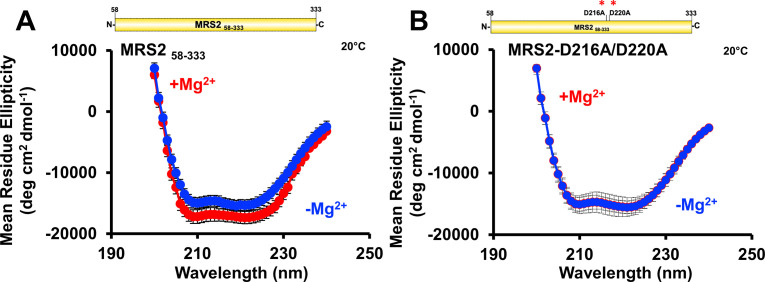
Secondary structure of MRS2_58–333_ and MRS2_58–333_ D216A/D220A (NTD). **(A, B)** Far-UV CD spectra of (A) MRS258–333 and (B) MRS258–333 D216A/D220A in the absence (blue) and presence (red) of 5 mM MgCl2. In (A, B), data are means ± SEM of n = 3 experiments with samples from three protein preparations. Far-UV CD spectra were acquired with 0.5 mg/ml protein in 20 mM Tris, 150 mM NaCl, and 1 mM DTT, pH 8.0, at 20°C.

Having observed that Mg^2+^ binding affects the quaternary and tertiary levels of MRS2 NTD structure, we next used far-UV circular dichroism (CD) spectroscopy to assess the secondary structure. At 37°C, MRS2_58–333_ displayed well-defined mean residue ellipticity minima at ∼208 and ∼222 nm, indicating high levels of α-helicity (Fig 8A). Remarkably, addition of 5 mM MgCl_2_ directly to the cuvette resulted in an increase in α-helicity, evidenced by more intense negative ellipticity at ∼208 and 222 nm (Fig 8A and C). Similar results were observed for MRS2_58–333_ at 20°C (Fig S4A).

To gain further evidence that D216 and D220 play a critical role in Mg^2+^ binding to the NTD, we also acquired far-UV CD spectra using D216A/D220A MRS2_58–333_. The far-UV CD spectrum of the double mutant showed a similar level of negative ellipticity as WT with two well-defined minima at ∼208 and ∼222 nm (Fig 8A and B), suggesting that secondary structure folding was not perturbed by the D216A/D220A substitutions. Unlike WT, adding 5 mM MgCl_2_ directly to the cuvette did not significantly alter the ellipticity for the double mutant (Fig 8D). Unchanging spectra after 5 mM MgCl_2_ addition were also observed for the double mutant at 20°C (Fig S4B).

We next evaluated thermal stability by monitoring the change in far-UV CD ellipticity at 222 nm as a function of increasing temperature. The thermal melts of MRS2_58–333_ acquired in the absence of Mg^2+^ exhibited a mean Boltzmann sigmoidal–fitted midpoint of temperature denaturation (T_m_) of 51 ± 0.62 °C (Fig 8E). Protein samples supplemented with 5 mM MgCl_2_ were stabilized by ∼7°C as the mean T_m_ shifted to 58 ± 0.36°C (Fig 8E). Thermal melt experiments with the D216A/D220A MRS2_58–333_ protein revealed similar mean T_m_ values of 52 ± 0.70°C and 52 ± 0.64°C in the presence and absence of Mg^2+^, respectively (Fig 8F).

Collectively, these data reveal that Mg^2+^ binding stabilizes the MRS2 NTD, consistent with an observed increase in α-helicity. Furthermore, the structural and stability augmentation is dependent on D216 and D220 as mutation of these residues renders the NTD insensitive to Mg^2+^, reinforcing the importance of these sites to coordinating Mg^2+^.

### Mg^2+^ binding to the MRS2 NTD negatively regulates mitochondrial Mg^2+^ uptake

**Figure 9.**
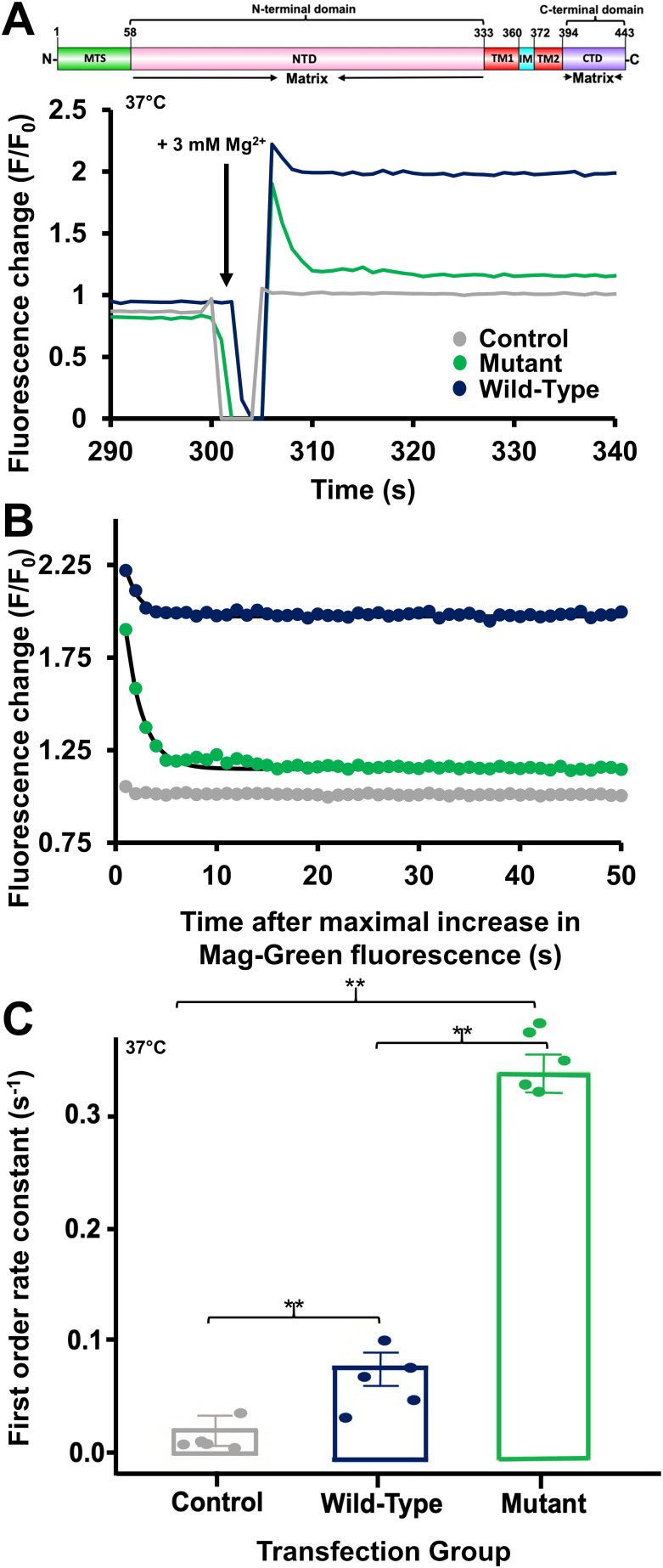
Mitochondrial Mg^2+^ uptake rates in HeLa cells overexpressing WT or D216A/D220A human MRS2. **(A)** Representative Mag-Green fluorescence traces reporting relative extramitochondrial Mg^2+^ before and after 3 mM MgCl_2_ addback to the extracellular bath at 300 s (black arrows) for control-, WT MRS2–, and MRS2 D216A/D220A–transfected cells. **(A, B)** Representative single exponential fits to the Mag-Green fluorescence decays after the 3 mM MgCl_2_ addback shown in (A), reporting Mg^2+^ clearance and taken as a measure of mitochondrial Mg^2+^ uptake. **(C)** One-way ANOVA followed by Tukey’s post hoc comparison of mitochondrial Mg^2+^ uptake rates for control-, WT MRS2–, and MRS2 D216A/D220A–transfected cells, where *P < 0.05, **P < 0.01, and ***P < 0.001. In (A, B, C), data were acquired at 37°C and were normalized as F/F_0_, where F is the Mag-Green fluorescence at any time point and F_0_ is the mean 30 s baseline fluorescence before the addition of EDTA/digitonin, and control, WT, and D216A/D220A data are coloured grey, black, and green, respectively.

**Figure S5.**
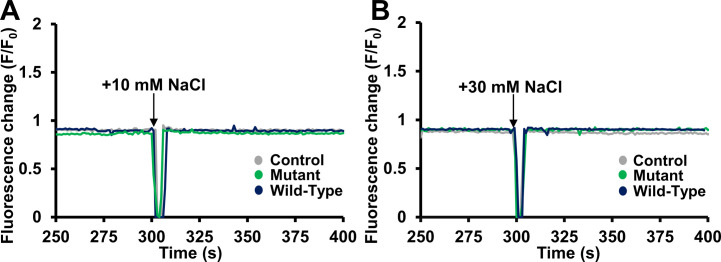
Mag-Green fluorescence changes in permeabilized HeLa cells in response to increases in osmolarity. **(A)** Mag-Green fluorescence traces before and after 10 mM NaCl addback to the extracellular bath at 300 s (black arrows) for control-, WT MRS2-, and MRS2 D216A/D220A-transfected cells. **(B)** Mag-Green fluorescence traces before and after 30 mM NaCl addback to the extracellular bath at 300 s (black arrows) for control-, WT MRS2-, and MRS2 D216A/D220A-transfected cells. In (A, B), data were acquired at 37°C and were normalized as F/F0 where F is the Mag-Green fluorescence at any time point, and F0 is the mean 30 s baseline fluorescence before the addition of EDTA/digitonin, and control, WT, and D216A/D220A data are coloured grey, black and green, respectively. Traces are an average of three technical replicates for each transfection group.

To link our in vitro observations with MRS2 function, we monitored Mg^2+^ dynamics using Mag-Green in HeLa cells overexpressing WT and D216A/D220A MRS2. HeLa cells were incubated with the membrane-permeant Mag-Green-AM to cytosolically load the cells with the Mg^2+^ sensitive dye. After washing and bathing the cells with intracellular buffer (IB), the plasma membrane (PM) was permeabilized with 5 μM digitonin, and 3 mM MgCl_2_ was added to the bath. Mitochondrial Mg^2+^ uptake rates were inferred from the clearance of extramitochondrial Mg^2+^, measured as the decrease in Mag-Green fluorescence, as previously done (Daw et al, 2020). After MgCl_2_ addback, digitonin-permeabilized HeLa cells transfected with empty pCMV vector (control), pBSD-MRS2 (WT), and pBSD-MRS2 D216A/D220A (mutant), all showed increases in Mag-Green fluorescence followed by a decay associated with Mg^2+^ clearance (Fig 9A). Fitting the data to single exponential decays indicated greater extramitochondrial Mg^2+^ clearance rates for WT MRS2-expressing cells compared with control cells and mutant MRS2-expressing cells compared with control and WT MRS2-expressing cells (Fig 9B and C). Addition of 10 mM or 30 mM NaCl to similarly permeabilized cells caused no change in the Mag-Green signal, suggesting minimal influence of osmolarity on our Mag-Green measurements (Fig S5A and B).

Collectively, these data suggest that MRS2 overexpression enhances extramitochondrial Mg^2+^ clearance, and Mg^2+^ interactions with the MRS2 NTD act as a negative feedback switch to temper Mg^2+^ uptake into the mitochondria.

### Gain of function D216K/D220K mutant relieves negative feedback on MRS2 activity

**Figure 10.**
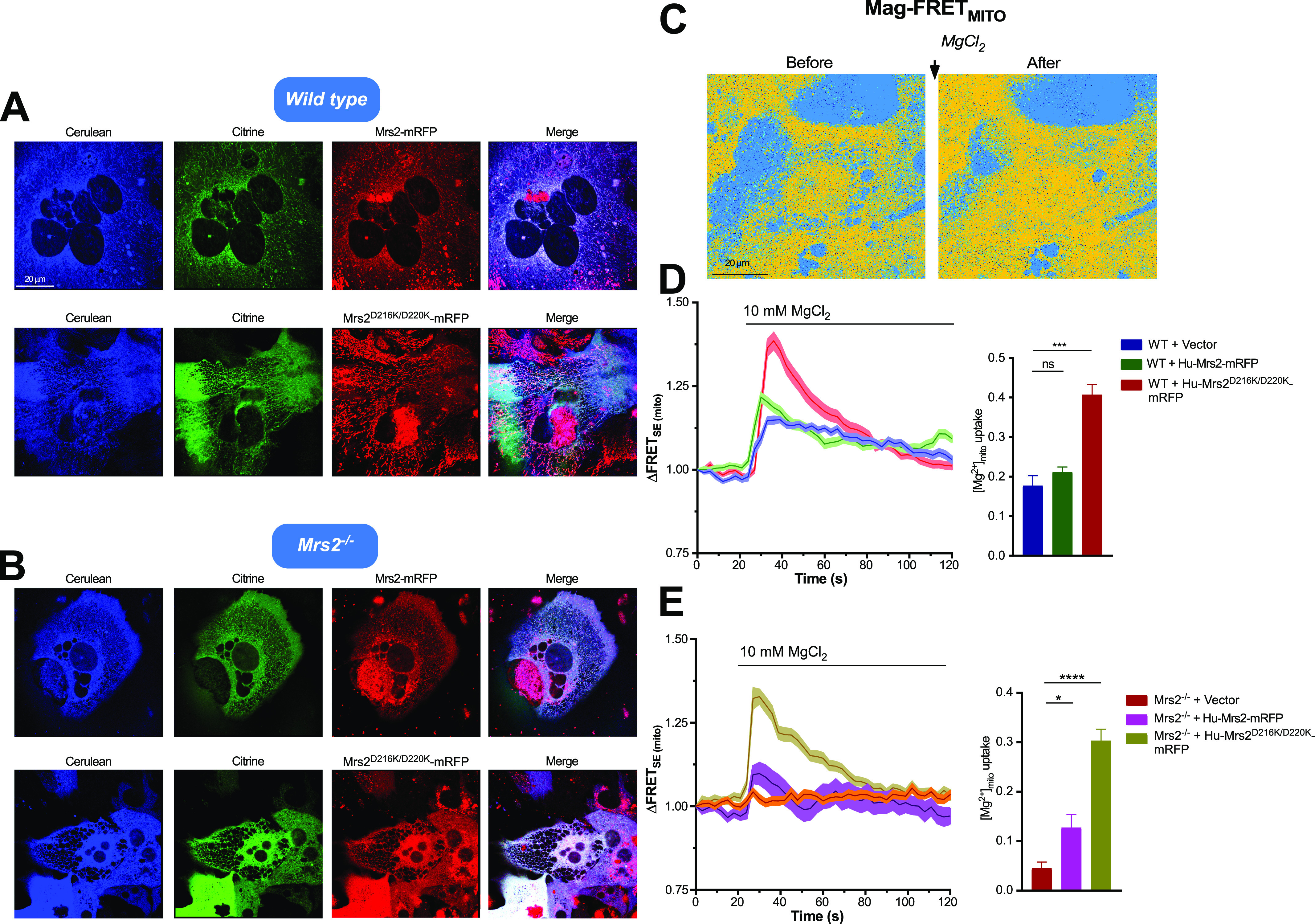
D216K/D220K double mutant enhances human MRS2 channel activity. **(A, B)** Representative confocal images showing expression and mitochondrial localization of human WT MRS2-mRFP and MRS2 D216K/D220K-mRFP in WT (A) or Mrs2^−/−^ (B) primary murine hepatocytes transduced with the adenoviral mito-Mag-FRET sensor. **(C)** Representative FRET images before and after 10 mM MgCl_2_ addition to primary hepatocytes expressing mito-Mag-FRET. **(D)** Mean traces (left panel) showing relative mito-Mag-FRET ratio changes upon addition of 10 mM MgCl_2_ and comparisons of peak mitochondrial Mg^2+^ uptake responses in WT hepatocytes (right panel). **(E)** Mean traces (left panel) showing relative mito-Mag-FRET ratio changes upon addition of 10 mM MgCl_2_ and comparisons of peak mitochondrial Mg^2+^ uptake responses in Mrs2^−/−^ hepatocytes (right panel). In (A, B, C, D, E), all measurements were performed at 37°C and quantified values are normalized as FRET/FRET_0_, where FRET and FRET_0_ are the signals at any time and initial timepoints, respectively. Unpaired *t* test was performed for comparison of peak responses, where *P < 0.05, ***P < 0.001, ****P < 0.0001; ns, not significant. Data are means ± SEM of n = 3 for WT + vector, n = 5 for WT + Hu-MRS2-mRFP, n = 3 for WT + Hu-MRS2^Δ216K/D220K^-mRFP, n = 3 for Mrs2^−/−^ + vector, n = 4 for Mrs2^−/−^ + Hu-MRS2-mRFP, and n = 3 for Mrs2^−/−^ + Hu-MRS2^Δ216K/D220K^-mRFP, where n is the number of separate transfections.

**Figure S6.**
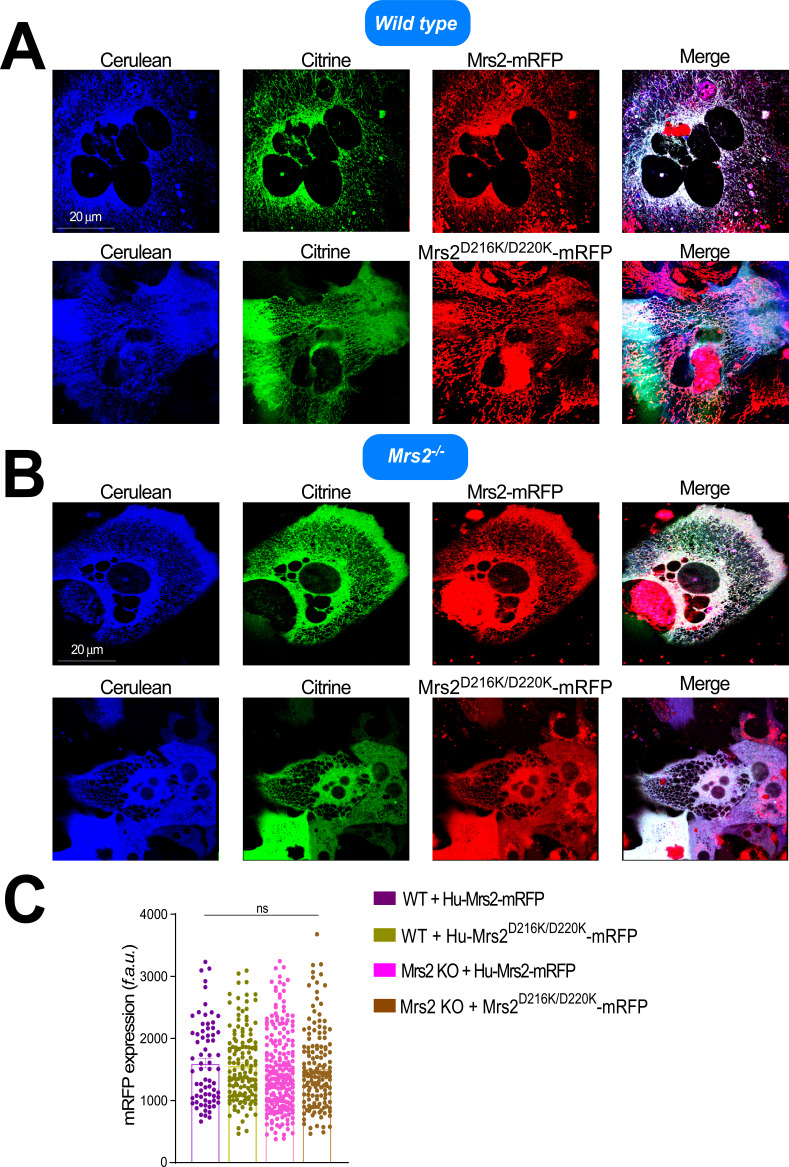
Ectopic expression of human WT and D216K/D220K MRS2 in murine hepatocytes. **(A)** High-resolution representative confocal images, showing expression and mitochondrial localization of human WT MRS2-mRFP in WT murine hepatocytes transduced with the adenoviral mito-Mag-FRET sensor. **(B)** High-resolution representative confocal images showing expression and mitochondrial localization of human D216K/D220K MRS2-mRFP in Mrs2−/− murine hepatocytes transduced with the adenoviral mito-Mag-FRET sensor. **(C)** Human WT MRS2-mRFP and D216K/D220K MRS2-mRFP fluorescence intensity level comparisons after expression in WT and Mrs2−/− murine hepatocytes. Data were compared using one-way ANOVA after Tukey’s post-hoc test.

**Figure S7.**
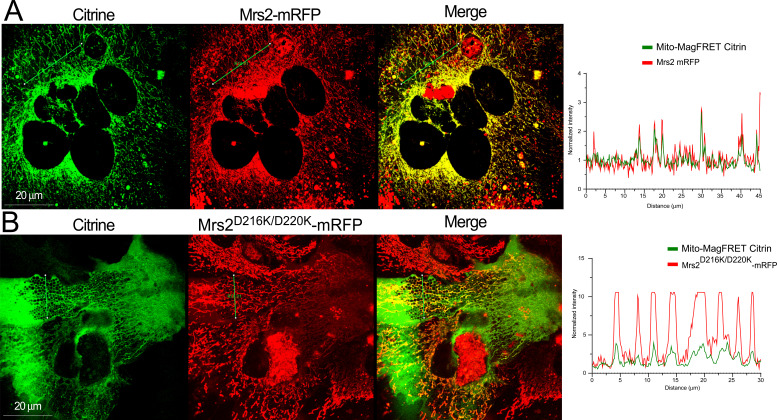
Co-localization of human WT and D216K/D220K MRS2 with mito-Mag-FRET in murine hepatocytes. **(A)** Line scan intensity profile of the mito-Mag-FRET citrine and human WT MRS2-mRFP fluorescence signals in WT murine hepatocytes. At left, the location of the line (green), where intensity was evaluated is shown in the citrine, mRFP, and merged channels. At right, the normalized pixel intensity over the distance of the line is shown for the mRFP (red) and citrine (green) channels. **(B)** Line scan intensity profile of the mito-Mag-FRET citrine and human D216K/D220K MRS2-mRFP fluorescence signals in WT murine hepatocytes. At left, the location of the line (green), where intensity was evaluated is shown in the citrine, mRFP, and merged channels. At right, the normalized pixel intensity over the distance of the line is shown for the for the mRFP and citrine channels.

To probe whether mutation of the Mg^2+^-binding site causes a bona fide gain of function, we reconstituted human WT and D216K/D220K MRS2 in WT and Mrs2 knockout (Mrs2^−/−^) hepatocytes. Primary murine hepatocytes were transfected with empty vector, human MRS2-mRFP, or MRS2 D216K/D220K-mRFP plasmids. 24 h post-transfection, a genetically encoded, mitochondrially targeted Mag-FRET biosensor (i.e., mito-Mag-FRET) was transduced into the cells to directly measure mitochondrial Mg^2+^ uptake. This mito-Mag-FRET sensor was previously shown to localize to hepatocyte mitochondria, reporting reciprocal lactate-induced Mg^2+^ responses compared with an ER-targeted/retained Mag-FRET sensor (Daw et al, 2020). Murine Mrs2-mRFP under the control of a CMV promoter, similar to the construct used in the present study, was also shown to properly co-localize with dihydrorhodamine-123 in hepatocyte mitochondria (Daw et al, 2020). Here, confocal images of the transfected/transduced WT hepatocytes show strong co-expression and co-localization of MRS2 and MRS2 D216K/D220K with the cerulean and citrine fluorescence of the mito-Mag-FRET biosensor, indicating mitochondrial localization of the human WT and mutant MRS2 in murine cells (Figs 10A and S6A). The pixel intensity profiles of the mito-Mag-FRET citrine and human MRS2-mRFP (WT and mutant) signals exhibit coincident peak maxima, consistent with this co-localization (Fig S7A and B). Human WT and mutant MRS2 also strongly co-localized with the mito-Mag-FRET fluorophores in the Mrs2^−/−^ hepatocytes (Figs 10B and S6B).

As expected, a 10-mM MgCl_2_ bolus increased the mito-Mag-FRET signal in WT cells (Fig 10C). Although WT hepatocytes expressing human MRS2 showed a similar mito-Mag-FRET response to the MgCl_2_ bolus, cells transfected with human MRS2 D216K/D220K exhibited highly potentiated mitochondrial Mg^2+^ uptake compared with controls (Fig 10D). Human MRS2 was fully capable of functionally reconstituting the Mg^2+^ channel in Mrs2^−/−^ hepatocyte mitochondria. Remarkably, the MRS2 D216K/D220K formed channels that greatly enhanced mitochondrial Mg^2+^ uptake compared with WT human MRS2 (Fig 10E). Note that the mRFP fluorescence intensities of human WT MRS2 and human MRS2 D216K/D220K in WT and Mrs2^−/−^ hepatocytes were similar, suggesting comparable expression levels across all groups (Fig S6C). Given the striking potentiation of Mg^2+^ uptake in hepatocytes co-expressing the D216K/D220K mutant but not WT human MRS2 with endogenous Mrs2, our data suggest that the Mg^2+^ binding–deficient MRS2 mutant dominantly mediates a gain of mitochondrial Mg^2+^ uptake function.

## Discussion

Human MRS2 belongs to the heterogeneous CorA/Mrs2/Alr1 superfamily of Mg^2+^ transporters, where CorA, Alr1, and Mrs2/MRS2 comprise the principal Mg^2+^ uptake systems in bacteria, yeast PM, and mitochondria, respectively. Bacterial CorA has been the most extensively studied family member, yielding mechanistic and functional insights on these channels (Franken et al, 2022; Jin et al, 2022). Nevertheless, given the low sequence similarity between human MRS2 and these homologues, there remains a major knowledge gap concerning the precise structural, functional, and regulatory mechanisms of human MRS2. Here, we isolated and biophysically characterized the largest domain of human MRS2, corresponding to the matrix-oriented NTD. We found that MRS2 NTD forms a homodimer under dilute conditions, which may be a building block to higher order oligomers. Remarkably, Mg^2+^ and Ca^2+^ disassembled both higher order MRS2 NTD oligomers and homodimers but not full-length MRS2 assemblies. In contrast, Co^2+^ disassembled full-length MRS2 oligomers but not MRS2 NTD. We estimated the K_d_ of Mg^2+^ binding to be ∼0.14 mM, and a D216A/D220A MRS2 NTD double mutant disrupted this Mg^2+^ binding but had no effect on Ca^2+^ binding, indicating disparate binding sites for these two divalent cations. Remarkably, this D216A/D220A double mutant abrogated the enhanced solvent exposed hydrophobicity, α-helicity, and thermal stability mediated by Mg^2+^ binding. Furthermore, MRS2 NTD oligomers and homodimers harboring this double mutation remained intact in the presence of Mg^2+^. Finally, we showed that reconstitution of D216A/D220A or D216K/D220K MRS2 mutants in mammalian cells greatly increased mitochondrial Mg^2+^ uptake compared with WT MRS2-expressing cells.

**Figure 11.**
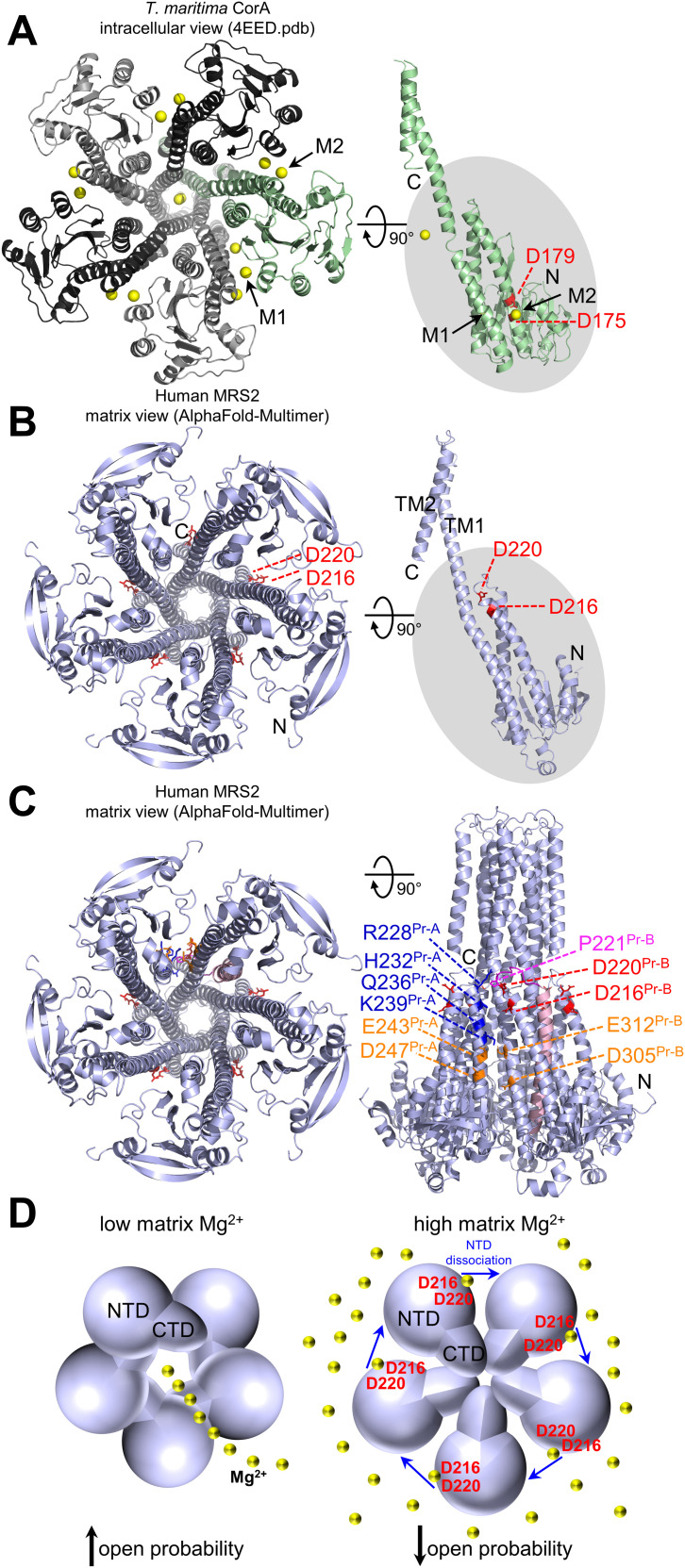
Model of Mg^2+^-induced negative feedback in MRS2 function. **(A)** At left, intracellular view of the experimentally determined *T*. *maritima* CorA pentamer structure. A backbone cartoon representation of each protomer is shown with M1 and M2 Mg^2+^-binding sites indicated (black arrows). At right, view of a single protomer (green), rotated 90° relative to the pentamer view. The D175 and D179 of the bacterial DALVD are coloured red. **(B)** At left, matrix view of the AlphaFold-Multimer model of a human MRS2 homopentamer. At right, view of a single protomer (light blue) rotated 90° relative to the pentamer view. The D216 and D220 positions of the human DALVD are coloured red. **(C)** At left, the matrix view AlphaFold-Multimer model of a human MRS2 homopentamer. At right, view of the 90° rotated homopentamer, highlighting the position of the D216 and D220 Mg^2+^ binding residues (red), the adjacent loop (magenta), interprotomer P221 (magenta):R228 (blue) contacts, connected helix-mediating additional interprotomer contacts (light pink), additional potential metal-binding sites across protomers involving E243, D247, E312, and D305 (orange) and the location of H232, Q236, and K239 (blue) on an adjacent protomer near D216 and D220. Pr-A, protomer-A; Pr-B, protomer-B. **(D)** Model of matrix Mg^2+^-induced inhibition of human MRS2 open probability. At low matrix Mg^2+^ (left), the human MRS2 channel has a high open probability; at high matrix Mg^2+^ (right), Mg^2+^ binding to a site mediated by D216 and D220 dissociates NTD complexes, leading to a low open probability. Despite weakened NTD interactions at high Mg^2+^, the channel remains assembled via TM and/or CTD interactions. A pentameric human MRS2 channel is depicted in homology to CorA, but the functional stoichiometry of the human MRS2 channel remains unknown. In (A, B, C), the same relative view is highlighted by the grey oval, Mg^2+^ ions are represented by yellow spheres and the amino and carboxyl termini are labeled N and C, respectively. In (B, C), the DALVD and adjacent loop sequence show AlphaFold2 pLDDT confidence scores of < 90 and < 70, indicating the predicted orientations of the side chains and backbone, respectively, are not reliable. An experimentally determined human MRS2 structure is needed to firmly establish the precise stoichiometry and the positions of the D216 and D220 side chains as well as nearby residues with respect to the assembled channel. Source data are available for this figure.

Several CorA crystal and cryoelectron microscopy structures have been elucidated in the presence of divalent cations, revealing a pentameric assembly (Eshaghi et al, 2006; Payandeh & Pai, 2006; Guskov et al, 2012; Pfoh et al, 2012; Nordin et al, 2013; Cleverley et al, 2015; Matthies et al, 2016; Johansen et al, 2022). The first TM, which lines the channel pore, and second TM orient the intervening GMN motif for ion binding and selectivity at the pore entrance (Pfoh et al, 2012). Upstream of TM1, a large intracellular domain of CorA, analogous to the matrix-oriented human MRS2 NTD, fans out into the cytoplasm and is composed of eight α-helices and a six-stranded β-sheet (*T*. *maritima*; 4EED.pdb) (Fig 11A). For *T*. *maritima* CorA, two Mg^2+^-binding sites (M1 and M2) have been identified per intracellular domain (Pfoh et al, 2012). M1 is made up of D89 and D253, whereas M2 is comprised of D175 and D179. Whereas earlier studies indicated that a symmetrizing of the pentameric intracellular domain assembly upon Mg^2+^ binding to the NTD closes the channel (Pfoh et al, 2012; Matthies et al, 2016), more recent work indicates both symmetric and asymmetric assemblies are formed in the presence and absence of Mg^2+^, and channel conductance is dependent on lowered symmetric state population and coupled with a reduced energy barrier to an ensemble of open states in low Mg^2+^ (Kowatz & Maguire, 2019; Johansen et al, 2022). Nevertheless, it is evident that Mg^2+^ binding increases the rigidity/decreases the dynamics of the CorA intracellular domain (Chakrabarti et al, 2010; Pfoh et al, 2012; Rangl et al, 2019; Johansen et al, 2022).

**Figure S8.**
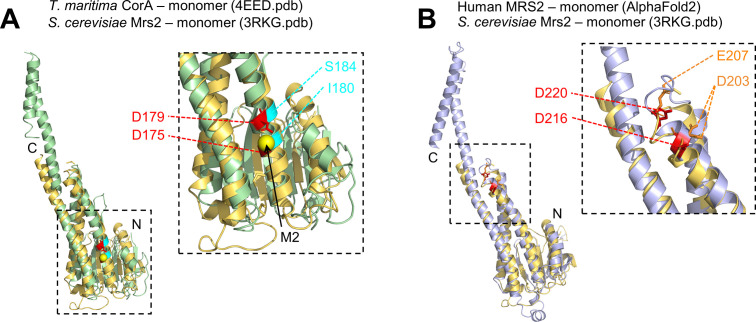
Three dimensional (3D) structural conservation of the DALVD region in yeast Mrs2. **(A)** 3D structural alignment of *T*. *maritima* CorA and the *S*. *cerevisiae* Mrs2 matrix domain showing the CorA M2 Mg^2+^ binding residues (D175 and D179; red sticks) are not conserved in the structurally analogous region in yeast, existing as I180 and S184 (cyan). The M2 Ca^2+^ ion is shown as a yellow sphere. **(B)** 3D structural alignment of the human MRS2 AlphaFold2 monomer model and the *S*. *cerevisiae* Mrs2 matrix domain suggesting the human Mg^2+^ binding residues we identified (D216 and D220; red sticks) are conserved in the structurally analogous region in yeast, existing as D203 and E207 (orange sticks). In (A, B), the 3D structural alignment was done using the TM-align server (Zhang & Skolnick, 2005) using 4EED.pdb, 3RKG.pdb, and AlphaFold2 monomer model (https://alphafold.ebi.ac.uk/entry/Q9HD23), coordinates for the bacterial, yeast, and human structures, respectively. The large dashed boxes at right show zoomed views of the corresponding smaller dashed boxes at left. The amino and carboxyl termini are labeled N and C, respectively.

The M1 Mg^2+^-binding site of *T*. *maritima* CorA does not appear conserved in human MRS2 based on multiple sequence alignments (Figs S1 and S2) or 3D superposition of the CorA crystal (Pfoh et al, 2012) and human AlphaFold2 (Jumper et al, 2021) MRS2-predicted structures. Furthermore, the AlphaFold2 model of human MRS2 orients the D216 and D220 Mg^2+^-binding residues, which we experimentally validated, six helical turns closer to the membrane domains compared with the CorA M2 DALVD residues (Fig 11A and B). Interestingly, a superposition of *T*. *maritima* CorA and *S*. *cerevisiae* Mrs2 (Khan et al, 2013) crystal structures structurally aligns the bacterial CorA DALVD with an INVMS sequence in *S*. *cerevisiae* Mrs2 (Fig S8A), suggesting the bacterial M2 Mg^2+^-binding site is not conserved in yeast. However, a superposition of the human AlphaFold2 model with the *S*. *cerevisiae* crystal structure suggests a structural conservation between human D216 and D220 and yeast D203 and E207 (of DLENE) (Fig S8B), not apparent from the multiple sequence alignments (Figs S1 and S2).

Although the stoichiometry of the human MRS2 channel remains unknown, we generated a homopentamer in homology to CorA, using AlphaFold-Multimer (Evans et al, 2022 *Preprint*) to model how Mg^2+^ binding to D216 and D220 may cause matrix domain disassembly (Fig 11B). PDBsum analysis (Laskowski et al, 2018) of the multimer model indicates that D216 and D220 do not participate in interprotomer H-bonding, salt-bridges, or other nonbonded contacts. However, P221 H-bonds and forms other nonbonded contacts with R228 of an adjacent subunit (Fig 11C). Furthermore, the loop following D220 exits into a 32-residue helix that contains many additional interprotomer contacts (Fig 11C). We showed that Mg^2+^ binding to D216 and D220 increases α-helicity, which could rearrange the adjacent loop that contains P221 and the position of the immediate downstream helix, leading to subunit dissociation. The human MRS2 homopentamer model also reveals clusters of negatively charged residues across interfaces (i.e., E243, D247, D305, and E312), which could mediate additional divalent cation–binding sites (Fig 11C). Ultimately, experimentally determined high-resolution structures of human MRS2 are needed to reveal channel stoichiometry, the basis for assembly and mechanisms for Mg^2+^-induced disassembly.

Mg^2+^ increased α-helicity and stability of the human MRS2 NTD, consistent with past NMR data, showing decreased backbone dynamics of CorA in the presence of high Mg^2+^ (Johansen et al, 2022). The far-UV CD spectra reported here resemble previous data from our laboratory, where we found no effect by MgCl_2_, likely due to variability in protein concentration measurements (Daw et al, 2020). Here, we applied MgCl_2_ addback to the same sample to expose the secondary structure change. Several lines of evidence suggest distinct Ca^2+^- and Mg^2+^-binding sites on the MRS2 NTD. First, the change in intrinsic fluorescence caused by the two cations was different; second, whereas Mg^2+^ increased, Ca^2+^ decreased solvent accessible hydrophobicity; third, D216A/D220A double mutant increased the Mg^2+^ K_d_ ∼sevenfold, whereas having no effect on the Ca^2+^ K_d_; finally, the Mg^2+^-dependent solvent accessible hydrophobicity change was abrogated, whereas the Ca^2+^ response was maintained by the D216A/D220A double mutant. Given the MM has a free Mg^2+^ concentration of ∼0.5–1.5 mM (Jung et al, 1990; Rutter et al, 1990), the Mg^2+^ K_d_ of ∼0.14 mM reported here would suggest the MRS2 structure, stability, and oligomerization would be sensitive to physiologically relevant fluctuations in Mg^2+^ levels within the matrix.

A broad range of free MM Ca^2+^ concentrations in mammalian cells have been reported, dependent on cell type, stimulus, and indicator; moreover, most estimates are < 100 µM (reviewed in Fernandez-Sanz et al [2019]), much lower than our MRS2 NTD Ca^2+^ K_d_ estimate of ∼1 mM. Hence, the MRS2 NTD would have to be positioned close to a Ca^2+^ channel pore to be affected, where local Ca^2+^ concentrations may approach the ∼mM range (Chad & Eckert, 1984; Bauer, 2001; Tadross et al, 2013). Directly assessing how Ca^2+^ binding affects MRS2 activity in cellulo is problematic due to the weak Ca^2+^ K_d_ of 1 mM. For example, 100 μM matrix Ca^2+^ would occupy < 10% of the MRS2 Ca^2+^-binding sites and perturb mitochondrial membrane potential. Nevertheless, indirectly, mitochondrial Ca^2+^ uniporter KO studies show unaltered lactate-stimulated mitochondrial Mg^2+^ uptake (Daw et al, 2020), suggesting Ca^2+^ may not play a crucial role in MRS2 regulation.

Interestingly, although Co^2+^ dissociated larger full-length MRS2 assemblies, we observed no effect on MRS2 NTD by DLS. In contrast, Mg^2+^ and Ca^2+^ did not alter the assembly of full-length MRS2 but dissociated the MRS2 NTD. We posit that MRS2_58–333_ (NTD) within MRS2 full-length undergoes disassembly in the presence of Mg^2+^ and Ca^2+^, whereas the C-terminal domain and/or TM regions remain interacting. Such a change would be undetectable by DLS, as the complex size would be unaffected. Furthermore, we believe Co^2+^-mediated disassembly occurs via binding to a region outside the NTD. Because estimates for the mitochondrial concentration of Co^2+^ range from ∼50 to 90 nM (Tapiero et al, 2003; Czarnek et al, 2015), the precise physiological significance of Co^2+^ interactions with any human MRS2 domain remains unclear.

Using permeabilized and intact cells, our data show that Mg^2+^ binding to the MRS2 NTD negatively regulates the channel. Permeabilized cells overexpressing the D216A/D220A double-mutant MRS2 cleared extramitochondrial Mg^2+^ at increased rates compared with WT MRS2-expressing cells. Furthermore, human WT and D216K/D220K MRS2 were fully capable of reconstituting functional MRS2 channels in intact primary murine Mrs2^−/−^ hepatocytes, with the double mutant causing highly potentiated Mg^2+^ uptake in Mrs2^−/−^ and WT mitochondria, indicative of gain of function activity. We do not believe that osmolarity changes because of MgCl_2_ addition influenced these trends since construct-specific responses were observed and 10 mM or 30 mM NaCl had no effect on the Mag-Green responses (Fig S5). Interestingly, a study using CorA harboring mutations aimed at disrupting Mg^2+^ binding to M1 showed WT-like ^63^Ni^2+^ transport (Kowatz & Maguire, 2019). Here, we focused on an M2-like cluster of residues because M1 does not appear to be conserved in human MRS2, discovering a robust, dominantly increased mitochondrial Mg^2+^ uptake upon disruption of Mg^2+^ binding to the NTD.

In conclusion, our work reveals the large NTD functions as a negative feedback regulator of human MRS2 channel function. We propose Mg^2+^ binding to the MRS2 NTD, contributed by D216 and D220, disrupts NTD:NTD interactions without disassembly of the channel (Fig 11D). Mg^2+^ binding to the MRS2 NTD increases α-helicity, stability, and solvent exposed hydrophobicity but dissociates NTD:NTD complexes, which we believe underlie key structural changes that propagate to the pore and/or crucial gating residues to inhibit the channel (Fig 11D). These data distinguish human MRS2 from bacterial CorA observations, where Mg^2+^ binding to the analogous intracellular domain shields electrostatically repulsive interfaces, promoting and bridging a symmetric interaction between intracellular domains (Pfoh et al, 2012; Matthies et al, 2016).

Because D216 and D220 are predicted to be near H232, Q236, and K239 of an adjacent protomer (Fig 11C), the D216A/D220A mutation could potentially perturb inter–protomer interactions involving these residues. In this scenario, destabilization of inter-domain interactions could lead to increased human MRS2 channel open probability, similar to the Mg^2+^ dissociation-dependent mechanism recently articulated in detail for *T*. *maritima* CorA by the A. Guskov group (Nemchinova et al, 2021). However, this scenario appears to be inconsistent with the Mg^2+^-binding–induced monomerization we observed for the human MRS2 matrix domain and our observations that D216A/D220A does not alter the dimer stoichiometry of the human MRS2 matrix domain or the higher order full-length human MRS2 assembly in CHAPS micelles.

## Materials and Methods

### MRS2 expression and purification

The human MRS2 NTD was identified as residues 58–333 using bioinformatic identification of the mitochondrial targeting sequence (Fukasawa et al, 2015; Almagro Armenteros et al, 2019; Buchan & Jones, 2019), and TM1 and TM2 (Krogh et al, 2001), after the comparison with these predictions with the annotations in UniProt (Accession Q9HD23). Human MRS2_58–333_ was subcloned out of the BDS vector into pET-28a (Novagen) using PCR and NdeI and XhoI restriction sites. Overnight protein expression at 37°C from the pET-28a-MRS2_58–333_ vector was done using BL21 (DE3) *Escherichia coli* cells cultured in Luria broth, induced with 0.4 mM IPTG. Protein was purified under native conditions using HisPur (Thermo-Fisher Scientific) nickel–nitrilotriacetic acid beads as per the manufacturer guidelines. The wash and elution buffers contained 20 mM Tris (pH 8.0), 150 mM NaCl, 1 mM DTT, 20 mM Tris (pH 8.0), 150 mM NaCl, 1 mM DTT, and 300 mM imidazole. After dialysis in 20 mM Tris (pH 8.0), 150 mM NaCl, and 1 mM DTT buffer using a 3,500 D MWCO membrane (Thermo Fisher Scientific), the N-terminal hexa-histidine tag was cleaved with ∼2 U of bovine thrombin (Sigma-Aldrich) per 1 mg of protein. A final SEC step through an S200 10/300 Gl column (Cytiva), achieved >95% protein purity as assessed by SDS–PAGE and Coomassie blue staining.

D216A and D220A mutant were introduced into MRS2_58–333_ by PCR-mediated site-directed mutagenesis and expression and purification for this construct were performed as described for WT-MRS2_58–333_. The complementary mutagenic primers were 5′-CCTTGAGACCTTGGCTGCTTTGGTGGCCCCCAAACATTCTTC-3′ and 3′-GAAGAATGTTTGGGGGCCACCAAAGCAGCCAAGGTCTCAAGG-5′.

Full-length human MRS2 taken as residues 58–443 (MRS2_58–443_) was cloned and expressed using the same approach described for MRS2_58–333_. Purification was performed as described for the NTD, except with the addition of 10 mM CHAPS to both the elution and SEC buffers.

### SEC with in-line multi angle light scattering

SEC-MALS was performed using a Superdex 200 Increase 10/300 Gl column (Cytiva) connected to an AKTA pure FPLC system (Cytiva). A DAWN HELEOS II detector (Wyatt) and an Optilab TrEX differential refractometer (Wyatt) were used to estimate the molecular weight of MRS2_58–333_ under various experimental conditions. The entire in-line FPLC/MALS system was housed in cold cabinet maintained at ∼10°C. Data were obtained for four different protein concentrations: 0.45 mg/ml, 0.90 mg/ml, 2.5 mg/ml, and 5 mg/ml in 20 mM Tris (pH 8), 150 mM NaCl, and 1 mM DTT, using 100 µl injections of sample at each concentration. MALS molecular weights were determined in the accompanying ASTRA software (version 7.1.4; Wyatt) using Zimm plot analysis and a protein refractive index increment (dn/dc) = 0.185 L/g. Divalent cation containing experiments were performed by supplementing the running buffers and protein samples with 5 or 10 mM MgCl_2_ and CaCl_2_, as indicated.

### DLS

DLS measurements were made with a DynaPro NanoStar (Wyatt) instrument using a scattering angle of 90°. After centrifugation at 15,000*g* for 5 min at 4°C, 5 µl of supernatant was loaded into a JC-501 microcuvette, and measurements were collected as 10 consecutive acquisition scans with each acquisition being an average of 5 s. MRS2_58–333_ protein samples were assessed at 1.25 mg/ml in 20 mM Tris (pH 8), 150 mM NaCl, and 1 mM DTT in the absence or presence of 5 mM MgCl_2_, CaCl_2_, or CoCl_2_. Similarly, MRS2_58-443_ protein samples were assessed at 0.5 mg/ml in 20 mM Tris (pH 8), 150 mM NaCl, 10 mM CHAPS, and 1 mM DTT, in the absence or presence of 5 mM MgCl_2_, CaCl_2_, or CoCl_2_. For both proteins, data were acquired at 20 and 37°C, as indicated. All autocorrelation functions were deconvoluted using the regularization algorithm to extract the polydisperse distribution of hydrodynamic radii (R_h_) and cumulants fit for monodisperse weight-averaged R_h_ using the accompanying Dynamics software (version 7.8.1.3; Wyatt).

### Intrinsic fluorescence measurements for cation binding

A Cary Eclipse spectrofluorimeter (Agilent/Varian) was used to acquire intrinsic fluorescence emission spectra. Spectra were acquired for 0.1 mg/ml MRS2_58–333_ in 20 mM Tris (pH 8), 150 mM NaCl, and 1 mM DTT, using a 600-µl quartz cuvette. The fluorescence emission intensities were recorded at 22.5°C from 300 to 450 nm, using a 1 nm data pitch and an excitation wavelength of 280 nm. Excitation and emission slit widths were set to 5 and 10 nm, respectively, and the photomultiplier tube detector was set to 650 V. Emission spectra were obtained before and after supplementation with increasing concentrations of CaCl_2_, MgCl_2_, or CoCl_2_, added directly to the cuvette. A total of 15 emission spectra were acquired with increasing concentrations of divalent cation between 0 and 5 mM. Spectral intensities at 330 nm were corrected for the dilution associated with the volume change upon each addition to the cuvette, and resultant curves were fit to a one site binding model that takes into account protein concentration using R (version 4.2.1) to extract apparent equilibrium dissociation constants (K_d_).

### Extrinsic 8-anilinonapthalene-1-sulfonic acid fluorescence

Extrinsic ANS fluorescence measurements were performed using a Cary Eclipse spectrofluorometer (Agilent/Varian). Spectra were acquired at 15°C for 30 µM MRS2_58–333_ in 20 mM Tris (pH 8), 150 mM NaCl, 1 mM DTT, and 0.05 mM ANS, using a 600-µl quartz cuvette. The excitation wavelength was set to 372 nm, and the extrinsic ANS fluorescence emission spectra were acquired from 400 to 600 nm, with the photomultiplier tube detector set at 750 V. Excitation and emission slit widths were set to 10 and 20 nm, respectively, for all ANS experiments. To monitor divalent cation-induced changes in exposed hydrophobicity of MRS2_58–333_, 5 mM CaCl_2_ or 5 mM MgCl_2_ was added directly into the cuvette. Negligible effects of these cations on free ANS fluorescence were confirmed by acquiring similar spectra in the absence of protein.

### Far-UV CD spectroscopy

Far-UV CD spectra were acquired using a Jasco J-810 CD spectrometer with electronic Peltier temperature regulator (Jasco). Each spectrum was taken as an average of 3 accumulations, recorded at 37°C using a 1-mm path length quartz cuvette in 1-nm increments, 8-s averaging time, and 1 nm bandwidth. To eliminate technical variability in magnitude signals, after acquiring divalent cation-free spectra, 5 mM MgCl_2_ was added to the same samples, and spectra were re-acquired.

Thermal melts were recorded using a 1-mm path length quartz cuvette by monitoring the change in the CD signal at 222 nm from 20–95°C. A scan rate of 1°C min^−1^, 1 nm bandwidth, and 8-s averaging time was used during data acquisition. Mg^2+^-free and Mg^2+^-supplemented data were fit using a Boltzmann sigmoidal equation to estimate the midpoint of temperature denaturation (T_m_) using R (version 4.2.1).

### Mitochondrial Mg^2+^ uptake experiments using Mag-Green

HeLa cells were cultured in DMEM with high glucose (Wisent), 10% (vol/vol) FBS (Sigma-Aldrich), 100 μg/ml penicillin, and 100 U/ml streptomycin (Wisent) at 37°C in a 5% CO_2_, 95% (vol/vol) air mixture. Cells cultured in 35-mm dishes were transfected with PolyJet transfection reagent (SignaGen) according to the manufacturer guidelines. After ∼12 h, cells were incubated with 0.725 µM of the Mg^2+^ indicator Mag-Green for 30 min at 37°C. Cells were subsequently washed in divalent cation-free PBS, pH 7.4, and suspended in 2 ml of IB composed of 20 mM HEPES (pH 7), 130 mM KCl, 2 mM KH_2_PO_4_, 10 mM NaCl, 5 mM succinate, 5 mM malate, and 1 mM pyruvate. A 20% (vol/vol) cell suspension in IB was created in a quartz cuvette. Mag-Green fluorescence was monitored using a PTI QuantMaster spectrofluorimeter (Horiba) equipped with electronic temperature control using excitation and emission wavelengths of 506 and 531 nm, respectively, and excitation and emission slit widths of 2.5 and 2.5 nm, respectively. After a 30-s Mag-Green baseline fluorescence measurement, 2 mM EDTA plus 5 µM digitonin was added to permeabilize the PM. After 300 s, 3 mM MgCl_2_ (or 10–30 mM NaCl for osmolarity controls) was added to the cuvette and the Mag-Green signal was measured for 600 s. The first three intensity values recorded immediately after the cation addback were not included in any trace because of potential mixing and light artefact contributions. Mitochondrial Mg^2+^ uptake was correlated with the clearance of Mg^2+^, taken as the decrease in Mag-Green fluorescence after re-introduction of Mg^2+^ into the system, as previously done (Daw et al, 2020). The rates of Mag-Green fluorescence decrease were extracted by fitting the traces after the Mg^2+^ addback to a single exponential decay in R (version 4.2.1).

### Mito-Mag-FRET measurements in primary murine hepatocytes

Primary murine hepatocytes isolated from WT and Mrs2^−/−^ (Daw et al, 2020) mice grown on 25-mm collagen-coated glass coverslips were transfected with empty vector, human MRS2-mRFP (Hu-MRS2-mRFP), or Hu-MRS2 D216K/D220K-mRFP plasmids. 24 h post-transfection, hepatocytes were transduced with adenoviral mito-Mag-FRET (Daw et al, 2020) (20 MOI) for an additional 48 h. The subcellular localizations of ectopically expressing MRS2 and mito-Mag-FRET were visualized using a Leica SP8 confocal microscope. The cells were excited using the 405-nm laser line, and the emission was collected using the hybrid detector (HyD). The cerulean channel, 460–490 nm, and citrine channel, 510–550 nm, served to detect the emissions from the fluorescence resonance energy transfer (FRET). FRET emissions were acquired following donor and acceptor excitation sequences. Selected region of interests (ROIs) were drawn, and the acquired sequences were background corrected for acceptor cross excitation cross-talk, acceptor cross excitation, and FRET cross-talk (α = A/C; γ = B/C; δ = A/B). Time-lapse imaging was performed using the above-described acquisition mode, and the corresponding FRET efficiencies were analyzed. Selective ROIs focused on mitochondrial-targeted mito-Mag-FRET sensor signals, and the captured FRET sensitized emissions (relative FRET_SE_) were plotted.

To compare Hu-MRS2-mRFP and Hu-MRS2 D216/KD220K-mRFP protein abundance, the pixel intensities of mRFP signals were evaluated by selecting multiple ROIs for the following conditions: WT + Hu-MRS2-mRFP, WT + Hu-MRS2^D216K/D220K^-mRFP, Mrs2^−/−^ + Hu-MRS2-mRFP, and Mrs2^−/−^ + Hu-MRS2^D216K/D220K^-mRFP.

### Structure modeling and visualization

The human MRS2 (UniProt accession Q9HD23) homopentamer model was generated using AlphaFold-Multimer (v2.2.0) (Evans et al, 2022 *Preprint*) on the Shared Hierarchical Academic Research Computing Network (SHARCNET:www.sharcnet.ca) of the Compute Canada/Digital Research Alliance of Canada. A total of 25 predictions were made (five seeds per model), using a maximum template date of 2022-11-01 and all available genetic databases. The highest confidence homopentamer model based on predicted LDDT was taken for visualization and analysis. All structure images were generated using PyMOL (Version 2.4; Schrödinger, LLC.).

### Statistics

Unpaired *t* test was used when comparing two independent groups, paired *t* test was used when comparing outcomes of the same group before and after treatment, and one-way ANOVA after Tukey’s post hoc test was used for multiple means comparisons between three or more groups. All nonlinear regression fitting and statistical analyses were done in GraphPad Prism (4.03) or R (4.2.1).

## Data Availability

The AlphaFold-Multimer generated human MRS2 homopentamer coordinates have been included as Source Data, and all other data are available from the corresponding author upon request.

## Supplementary Material

Reviewer comments

## References

[bib1] Aden E, Carlsson M, Poortvliet E, Stenlund H, Linder J, Edstrom M, Forsgren L, Haglin L (2011) Dietary intake and olfactory function in patients with newly diagnosed Parkinson's disease: A case-control study. Nutr Neurosci 14: 25–31. 10.1179/174313211X12966635733312 21535918

[bib2] Almagro Armenteros JJ, Tsirigos KD, Sonderby CK, Petersen TN, Winther O, Brunak S, von Heijne G, Nielsen H (2019) SignalP 5.0 improves signal peptide predictions using deep neural networks. Nat Biotechnol 37: 420–423. 10.1038/s41587-019-0036-z 30778233

[bib3] Barbiroli B, Martinelli P, Patuelli A, Lodi R, Iotti S, Cortelli P, Montagna P (1999) Phosphorus magnetic resonance spectroscopy in multiple system atrophy and Parkinson's disease. Mov Disord 14: 430–435. 10.1002/1531-8257(199905)14:3<430::aid-mds1007>3.0.co;2-s 10348465

[bib4] Bauer PJ (2001) The local Ca concentration profile in the vicinity of a Ca channel. Cell Biochem Biophys 35: 49–61. 10.1385/cbb:35:1:49 11898855

[bib5] Berridge MJ, Lipp P, Bootman MD (2000) The versatility and universality of calcium signalling. Nat Rev Mol Cell Biol 1: 11–21. 10.1038/35036035 11413485

[bib6] Buchan DWA, Jones DT (2019) The PSIPRED protein analysis workbench: 20 years on. Nucleic Acids Res 47: W402–W407. 10.1093/nar/gkz297 31251384 PMC6602445

[bib7] Chad JE, Eckert R (1984) Calcium domains associated with individual channels can account for anomalous voltage relations of CA-dependent responses. Biophysical J 45: 993–999. 10.1016/s0006-3495(84)84244-7 PMC1434976 6329349

[bib8] Chaigne-Delalande B, Li FY, O’Connor GM, Lukacs MJ, Jiang P, Zheng L, Shatzer A, Biancalana M, Pittaluga S, Matthews HF, (2013) Mg^2+^ regulates cytotoxic functions of NK and CD8 T cells in chronic EBV infection through NKG2D. Science 341: 186–191. 10.1126/science.1240094 23846901 PMC3894782

[bib9] Chakrabarti N, Neale C, Payandeh J, Pai EF, Pomes R (2010) An iris-like mechanism of pore dilation in the CorA magnesium transport system. Biophys J 98: 784–792. 10.1016/j.bpj.2009.11.009 20197031 PMC2830438

[bib10] Clapham DE (2007) Calcium signaling. Cell 131: 1047–1058. 10.1016/j.cell.2007.11.028 18083096

[bib11] Cleverley RM, Kean J, Shintre CA, Baldock C, Derrick JP, Ford RC, Prince SM (2015) The Cryo-EM structure of the CorA channel from Methanocaldococcus jannaschii in low magnesium conditions. Biochim Biophys Acta 1848: 2206–2215. 10.1016/j.bbamem.2015.06.002 26051127 PMC4579555

[bib12] Czarnek K, Terpiłowska S, Siwicki AK (2015) Selected aspects of the action of cobalt ions in the human body. Cent Eur J Immunol 40: 236–242. 10.5114/ceji.2015.52837 26557039 PMC4637398

[bib13] Daw CC, Ramachandran K, Enslow BT, Maity S, Bursic B, Novello MJ, Rubannelsonkumar CS, Mashal AH, Ravichandran J, Bakewell TM, (2020) Lactate elicits ER-mitochondrial Mg(2+) dynamics to integrate cellular metabolism. Cell 183: 474–489.e17. 10.1016/j.cell.2020.08.049 33035451 PMC7572828

[bib14] de Baaij JHF, Hoenderop JGJ, Bindels RJM (2015) Magnesium in man: Implications for health and disease. Physiol Rev 95: 1–46. 10.1152/physrev.00012.2014 25540137

[bib15] Eshaghi S, Niegowski D, Kohl A, Molina DM, Lesley SA, Nordlund P (2006) Crystal structure of a divalent metal ion transporter CorA at 2.9 angstrom resolution. Science 313: 354–357. 10.1126/science.1127121 16857941

[bib16] Evans R, O’Neill M, Pritzel A, Antropova N, Senior A, Green T, Žídek A, Bates R, Blackwell S, Yim J, (2022) Protein complex prediction with AlphaFold-Multimer. BioRxiv. 10.1101/2021.10.04.463034 (Preprint posted March 10, 2022).

[bib17] Fernandez-Sanz C, De la Fuente S, Sheu SS (2019) Mitochondrial Ca(2+) concentrations in live cells: Quantification methods and discrepancies. FEBS Lett 593: 1528–1541. 10.1002/1873-3468.13427 31058316 PMC7573507

[bib18] Franken GAC, Huynen MA, Martinez-Cruz LA, Bindels RJM, de Baaij JHF (2022) Structural and functional comparison of magnesium transporters throughout evolution. Cell Mol Life Sci 79: 418. 10.1007/s00018-022-04442-8 35819535 PMC9276622

[bib19] Fukasawa Y, Tsuji J, Fu SC, Tomii K, Horton P, Imai K (2015) MitoFates: Improved prediction of mitochondrial targeting sequences and their cleavage sites. Mol Cell Proteomics 14: 1113–1126. 10.1074/mcp.m114.043083 25670805 PMC4390256

[bib20] Glancy B, Balaban RS (2012) Role of mitochondrial Ca^2+^ in the regulation of cellular energetics. Biochemistry 51: 2959–2973. 10.1021/bi2018909 22443365 PMC3332087

[bib21] Gregan J, Kolisek M, Schweyen RJ (2001) Mitochondrial Mg(2+) homeostasis is critical for group II intron splicing in vivo. Genes Dev 15: 2229–2237. 10.1101/gad.201301 11544180 PMC312778

[bib22] Guskov A, Eshaghi S (2012) The mechanisms of Mg^2+^ and Co^2+^ transport by the CorA family of divalent cation transporters. Curr Top Membr 69: 393–414. 10.1016/B978-0-12-394390-3.00014-8 23046658

[bib23] Guskov A, Nordin N, Reynaud A, Engman H, Lundback AK, Jong AJO, Cornvik T, Phua T, Eshaghi S (2012) Structural insights into the mechanisms of Mg^2+^ uptake, transport, and gating by CorA. Proc Natl Acad Sci U S A 109: 18459–18464. 10.1073/pnas.1210076109 23091000 PMC3494898

[bib24] Hartwig A (2001) Role of magnesium in genomic stability. Mutat Res 475: 113–121. 10.1016/s0027-5107(01)00074-4 11295157

[bib25] Jahnen-Dechent W, Ketteler M (2012) Magnesium basics. Clin Kidney J 5: i3–i14. 10.1093/ndtplus/sfr163 26069819 PMC4455825

[bib26] Jin F, Huang Y, Hattori M (2022) Recent advances in the structural biology of Mg(2+) channels and transporters. J Mol Biol 434: 167729. 10.1016/j.jmb.2022.167729 35841930

[bib27] Johansen NT, Bonaccorsi M, Bengtsen T, Larsen AH, Tidemand FG, Pedersen MC, Huda P, Berndtsson J, Darwish T, Yepuri NR, (2022) Mg(2+)-dependent conformational equilibria in CorA and an integrated view on transport regulation. Elife 11: e71887. 10.7554/elife.71887 35129435 PMC8865849

[bib28] Jumper J, Evans R, Pritzel A, Green T, Figurnov M, Ronneberger O, Tunyasuvunakool K, Bates R, Zidek A, Potapenko A, (2021) Highly accurate protein structure prediction with AlphaFold. Nature 596: 583–589. 10.1038/s41586-021-03819-2 34265844 PMC8371605

[bib29] Jung DW, Apel L, Brierley GP (1990) Matrix free magnesium changes with metabolic state in isolated heart mitochondria. Biochemistry 29: 4121–4128. 10.1021/bi00469a015 2361136

[bib30] Kanellopoulou C, George AB, Masutani E, Cannons JL, Ravell JC, Yamamoto TN, Smelkinson MG, Jiang PD, Matsuda-Lennikov M, Reilley J, (2019) Mg(2+) regulation of kinase signaling and immune function. J Exp Med 216: 1828–1842. 10.1084/jem.20181970 31196981 PMC6683994

[bib31] Khan MB, Sponder G, Sjoblom B, Svidova S, Schweyen RJ, Carugo O, Djinovic-Carugo K (2013) Structural and functional characterization of the N-terminal domain of the yeast Mg^2+^ channel Mrs2. Acta Crystallogr D Biol Crystallogr 69: 1653–1664. 10.1107/s0907444913011712 23999289

[bib32] Knoop V, Groth-Malonek M, Gebert M, Eifler K, Weyand K (2005) Transport of magnesium and other divalent cations: Evolution of the 2-TM-GxN proteins in the MIT superfamily. Mol Genet Genomics 274: 205–216. 10.1007/s00438-005-0011-x 16179994

[bib33] Kolisek M, Sponder G, Mastrototaro L, Smorodchenko A, Launay P, Vormann J, Schweigel-Rontgen M (2013) Substitution p.A350V in Na(+)/Mg(2)(+) exchanger SLC41A1, potentially associated with Parkinson's disease, is a gain-of-function mutation. PLoS One 8: e71096. 10.1371/journal.pone.0071096 23976986 PMC3744568

[bib34] Kowatz T, Maguire ME (2019) Loss of cytosolic Mg(2+) binding sites in the Thermotoga maritima CorA Mg(2+) channel is not sufficient for channel opening. Biochim Biophys Acta Gen Subj 1863: 25–30. 10.1016/j.bbagen.2018.09.001 30293964 PMC6342003

[bib35] Krogh A, Larsson B, von Heijne G, Sonnhammer EL (2001) Predicting transmembrane protein topology with a hidden markov model: Application to complete genomes. J Mol Biol 305: 567–580. 10.1006/jmbi.2000.4315 11152613

[bib36] Kumar A (2015) NMDA receptor function during senescence: Implication on cognitive performance. Front Neurosci 9: 473. 10.3389/fnins.2015.00473 26732087 PMC4679982

[bib37] Laskowski RA, Jablonska J, Pravda L, Varekova RS, Thornton JM (2018) PDBsum: Structural summaries of PDB entries. Protein Sci 27: 129–134. 10.1002/pro.3289 28875543 PMC5734310

[bib38] Lunin VV, Dobrovetsky E, Khutoreskaya G, Zhang R, Joachimiak A, Doyle DA, Bochkarev A, Maguire ME, Edwards AM, Koth CM (2006) Crystal structure of the CorA Mg^2+^ transporter. Nature 440: 833–837. 10.1038/nature04642 16598263 PMC3836678

[bib39] Marchi S, Pinton P (2014) The mitochondrial calcium uniporter complex: Molecular components, structure and physiopathological implications. J Physiol 592: 829–839. 10.1113/jphysiol.2013.268235 24366263 PMC3948548

[bib40] Matthies D, Dalmas O, Borgnia MJ, Dominik PK, Merk A, Rao P, Reddy BG, Islam S, Bartesaghi A, Perozo E, (2016) Cryo-EM structures of the magnesium channel CorA reveal symmetry break upon gating. Cell 164: 747–756. 10.1016/j.cell.2015.12.055 26871634 PMC4752722

[bib41] Merolle L, Sponder G, Sargenti A, Mastrototaro L, Cappadone C, Farruggia G, Procopio A, Malucelli E, Parisse P, Gianoncelli A, (2018) Overexpression of the mitochondrial Mg channel MRS2 increases total cellular Mg concentration and influences sensitivity to apoptosis. Metallomics 10: 917–928. 10.1039/c8mt00050f 29952392

[bib42] Moomaw AS, Maguire ME (2008) The unique nature of Mg^2+^ channels. Physiology (Bethesda) 23: 275–285. 10.1152/physiol.00019.2008 18927203 PMC2711038

[bib43] Nemchinova M, Melcr J, Wassenaar TA, Marrink SJ, Guskov A (2021) Asymmetric CorA gating mechanism as observed by molecular dynamics simulations. J Chem Inf Model 61: 2407–2417. 10.1021/acs.jcim.1c00261 33886304 PMC8154316

[bib44] Nordin N, Guskov A, Phua T, Sahaf N, Xia Y, Lu S, Eshaghi H, Eshaghi S (2013) Exploring the structure and function of Thermotoga maritima CorA reveals the mechanism of gating and ion selectivity in Co^2+^/Mg^2+^ transport. Biochem J 451: 365–374. 10.1042/bj20121745 23425532 PMC3629940

[bib45] Notredame C, Higgins DG, Heringa J (2000) T-Coffee: A novel method for fast and accurate multiple sequence alignment. J Mol Biol 302: 205–217. 10.1006/jmbi.2000.4042 10964570

[bib46] Oyanagi K, Kawakami E, Kikuchi-Horie K, Ohara K, Ogata K, Takahama S, Wada M, Kihira T, Yasui M (2006) Magnesium deficiency over generations in rats with special references to the pathogenesis of the Parkinsonism-dementia complex and amyotrophic lateral sclerosis of Guam. Neuropathology 26: 115–128. 10.1111/j.1440-1789.2006.00672.x 16708544

[bib47] Palombo I, Daley DO, Rapp M (2013) Why is the GMN motif conserved in the CorA/Mrs2/Alr1 superfamily of magnesium transport proteins? Biochemistry 52: 4842–4847. 10.1021/bi4007397 23781956

[bib48] Payandeh J, Pai EF (2006) A structural basis for Mg^2+^ homeostasis and the CorA translocation cycle. EMBO J 25: 3762–3773. 10.1038/sj.emboj.7601269 16902408 PMC1553185

[bib49] Pfoh R, Li A, Chakrabarti N, Payandeh J, Pomes R, Pai EF (2012) Structural asymmetry in the magnesium channel CorA points to sequential allosteric regulation. Proc Natl Acad Sci U S A 109: 18809–18814. 10.1073/pnas.1209018109 23112165 PMC3503211

[bib50] Pilchova I, Klacanova K, Tatarkova Z, Kaplan P, Racay P (2017) The involvement of Mg(2+) in regulation of cellular and mitochondrial functions. Oxid Med Cell Longev 2017: 6797460. 10.1155/2017/6797460 28757913 PMC5516748

[bib51] Piskacek M, Zotova L, Zsurka G, Schweyen RJ (2009) Conditional knockdown of hMRS2 results in loss of mitochondrial Mg^+^uptake and cell death. J Cell Mol Med 13: 693–700. 10.1111/j.1582-4934.2008.00328.x 18384665 PMC3822876

[bib52] Rangl M, Schmandt N, Perozo E, Scheuring S (2019) Real time dynamics of Gating-Related conformational changes in CorA. Elife 8: e47322. 10.7554/elife.47322 31774394 PMC6927688

[bib53] Romani A (2007) Regulation of magnesium homeostasis and transport in mammalian cells. Arch Biochem Biophys 458: 90–102. 10.1016/j.abb.2006.07.012 16949548

[bib54] Romani AM (2011) Cellular magnesium homeostasis. Arch Biochem Biophys 512: 1–23. 10.1016/j.abb.2011.05.010 21640700 PMC3133480

[bib55] Rutter GA, Osbaldeston NJ, McCormack JG, Denton RM (1990) Measurement of matrix free Mg^2+^ concentration in rat heart mitochondria by using entrapped fluorescent probes. Biochem J 271: 627–634. 10.1042/bj2710627 2244870 PMC1149608

[bib56] Schaffers OJM, Hoenderop JGJ, Bindels RJM, de Baaij JHF (2018) The rise and fall of novel renal magnesium transporters. Am J Physiol Renal Physiol 314: F1027–F1033. 10.1152/ajprenal.00634.2017 29412701

[bib57] Schlingmann KP, Weber S, Peters M, Niemann Nejsum L, Vitzthum H, Klingel K, Kratz M, Haddad E, Ristoff E, Dinour D, (2002) Hypomagnesemia with secondary hypocalcemia is caused by mutations in TRPM6, a new member of the TRPM gene family. Nat Genet 31: 166–170. 10.1038/ng889 12032568

[bib58] Sievers F, Wilm A, Dineen D, Gibson TJ, Karplus K, Li W, Lopez R, McWilliam H, Remmert M, Soding J, (2011) Fast, scalable generation of high-quality protein multiple sequence alignments using Clustal Omega. Mol Syst Biol 7: 539. 10.1038/msb.2011.75 21988835 PMC3261699

[bib59] Stryer L (1965) The interaction of a naphthalene dye with apomyoglobin and apohemoglobin. J Mol Biol 13: 482–495. 10.1016/s0022-2836(65)80111-5 5867031

[bib60] Tadross MR, Tsien RW, Yue DT (2013) Ca^2+^ channel nanodomains boost local Ca^2+^ amplitude. Proc Natl Acad Sci U S A 110: 15794–15799. 10.1073/pnas.1313898110 24019485 PMC3785779

[bib61] Tapiero H, Townsend DM, Tew KD (2003) Trace elements in human physiology and pathology. Copper. Biomed Pharmacother 57: 386–398. 10.1016/s0753-3322(03)00012-x 14652164 PMC6361146

[bib62] Trapani V, Wolf FI (2019) Dysregulation of Mg(2+) homeostasis contributes to acquisition of cancer hallmarks. Cell Calcium 83: 102078. 10.1016/j.ceca.2019.102078 31493712

[bib63] Waterhouse AM, Procter JB, Martin DMA, Clamp M, Barton GJ (2009) Jalview Version 2-a multiple sequence alignment editor and analysis workbench. Bioinformatics 25: 1189–1191. 10.1093/bioinformatics/btp033 19151095 PMC2672624

[bib64] Zhang Y, Skolnick J (2005) TM-Align: A protein structure alignment algorithm based on the TM-score. Nucleic Acids Res 33: 2302–2309. 10.1093/nar/gki524 15849316 PMC1084323

[bib65] Zhou H, Clapham DE (2009) Mammalian MagT1 and TUSC3 are required for cellular magnesium uptake and vertebrate embryonic development. Proc Natl Acad Sci U S A 106: 15750–15755. 10.1073/pnas.0908332106 19717468 PMC2732712

[bib66] Zsurka G, Gregan J, Schweyen RJ (2001) The human mitochondrial Mrs2 protein functionally substitutes for its yeast homologue, a candidate magnesium transporter. Genomics 72: 158–168. 10.1006/geno.2000.6407 11401429

