# Supplementary material for: The human MRS2 magnesium-binding domain is a regulatory feedback switch for channel activity
Source: Life Sci Alliance. 2023 Feb 8;6(4):e202201742. doi: 10.26508/lsa.202201742 (PMC9909464; doi:10.26508/lsa.202201742)
Supplement: Supplementary file 1 [file LSA-2022-01742_TableS1.docx]

**Table S1: Summary of SEC-MALS data for MRS2_58-333_.**

| **Protein** | **Protein concentration (mg/mL)** | **Cation**  **Concentration** | **Molecular Weight**  **(kDa) ^a^** | **Stoichiometric**  **Ratio ^b^** | **Elution**  **Volume (mL) ^c^** |
| --- | --- | --- | --- | --- | --- |
| **MRS2_58-333_** | 0.45 | 0 | 60.28 ± 2.58 | 1.86 | 14.84 |
|  |  | 5 mM MgCl_2_ | 31.32 ± 1.83 | 1.27 | 15.46 |
|  |  | 5 mM CaCl_2_ | 35.05 ± 0.64 | 1.08 | 15.62 |
|  | 0.90 | 0 | 58.53 ± 2.04 | 1.81 | 14.59 |
|  |  | 5 mM MgCl_2_ | 40.32 ± 7.98 | 1.24 | 15.53 |
|  |  | 5 mM CaCl_2_ | 31.59 ± 0.49 | 0.97 | 15.65 |
|  | 2.50 | 0 | 60.91 ± 1.82 | 1.92 | 14.85 |
|  |  | 5 mM MgCl_2_ | 29.35 ± 5.92 | 0.90 | 15.32 |
|  |  | 5 mM CaCl_2_ | 31.4 ± 1.00 | 0.94 | 15.38 |
|  | 5.00 | 0 | 61.28 ± 0.50 | 1.81 | 14.94 |
|  |  | 5 mM MgCl_2_ | 60.06 ± 0.47 | 1.93 | 14.66 |
|  |  | 5 mM CaCl_2_ | 28.60 ± 0.44 | 0.85 | 15.31 |
|  |  | 10 mM MgCl_2_ | 32.60 ± 0.44 | 0.88 | 15.34 |
|  |  | 10 mM CaCl_2_ | 34.57 ± 0.44 | 0.89 | 15.29 |
| **MRS2 D216A/D220A** | 2.50 | 0 | 59.73 ± 2.03 | 1.83 | 14.29 |
|  |  | 5 mM MgCl_2_ | 59.03 ± 2.38 | 1.82 | 14.35 |

^a^ SEC-MALS-determined molecular weight; errors (±) represent SEM of molecular weights determined for n=3 separate experiments from three protein preparations.

^b^ Stoichiometric ratio was calculated as the SEC-MALS-determined molecular weight divided by the theoretical monomeric molecular weight of MRS2_58-333_ (32.2 kDa).

^c^ Elution volume determined at peak maximum at 280 nm.
