# Supplementary material for: The human MRS2 magnesium-binding domain is a regulatory feedback switch for channel activity
Source: Life Sci Alliance. 2023 Feb 8;6(4):e202201742. doi: 10.26508/lsa.202201742 (PMC9909464; doi:10.26508/lsa.202201742)
Supplement: Supplementary file 2 [file LSA-2022-01742_TableS2.docx]

**Table S2: Summary of cumulants fits for MRS2_58-443_ and MRS2_58-333_.**

| **Protein** | **Temperature (***°***C)** | **Experimental Condition** | **Hydrodynamic Radius (nm) ^a^** | **Experimental Condition ^b^** | **Hydrodynamic Radius (nm) ^a^** |
| --- | --- | --- | --- | --- | --- |
| **MRS2_58-443_** | 20 | -MgCl_2_ | 10.58 ±1.04 | +MgCl_2_ | 10.68 ± 0.84 |
|  |  | -CaCl_2_ | 10.86 ±1.25 | +CaCl_2_ | 10.64 ± 0.95 |
|  |  | -CoCl_2_ | 10.97 ±1.38 | +CoCl_2_ | 2.43 ± 1.33 |
|  | 37 | -MgCl_2_ | 3.69 ± 0.50 | +MgCl_2_ | 3.47 ± 0.90 |
|  |  | -CaCl_2_ | 3.66 ± 0.78 | +CaCl_2_ | 3.44 ± 1.25 |
|  |  | -CoCl_2_ | 4.87 ± 0.95 | +CoCl_2_ | 2.59 ± 1.04 |
| **MRS2_58-333_** | 20 | -MgCl_2_ | 5.77 ± 0.88 | +MgCl_2_ | 5.83 ± 1.14 |
|  |  | -CaCl_2_ | 6.68 ± 0.85 | +CaCl_2_ | 4.55 ± 0.96 |
|  |  | -CoCl_2_ | 5.76 ± 0.50 | +CoCl_2_ | 5.83 ± 0.49 |
|  | 37 | -MgCl_2_ | 8.89 ± 0.46 | +MgCl_2_ | 4.95 ± 0.64 |
|  |  | -CaCl_2_ | 6.68 ± 0.70 | +CaCl_2_ | 4.55 ± 0.96 |
|  |  | -CoCl_2_ | 4.43 ± 0.53 | +CoCl_2_ | 4.17 ± 0.77 |
| **MRS2 D216A/D220A** | 20 | -MgCl_2_ | 5.69 ± 0.75 | +MgCl_2_ | 5.67 ± 0.74 |

^a^ Errors (±) are SEM from n=3 separate experiments from three protein preparations.

^b^ +divalent cation indicates supplementation with 5 mM of the indicated metal salt.
