# Supplementary material for: The human MRS2 magnesium-binding domain is a regulatory feedback switch for channel activity
Source: Life Sci Alliance. 2023 Feb 8;6(4):e202201742. doi: 10.26508/lsa.202201742 (PMC9909464; doi:10.26508/lsa.202201742)
Supplement: Supplementary file 3 [file LSA-2022-01742_TableS3.docx]

**Table S3: Summary of the fitted equilibrium dissociation constants (K_d_).**

| **Protein** | **Cation** | **K_d_ (mM) ^a^** | **Error ^b^** | **Avg. K_d_ (mM) ^c^** |
| --- | --- | --- | --- | --- |
| **MRS2_58-333_** | Mg^2+^ | 0.17 | ±0.07 | 0.14 ± 0.03 |
|  |  | 0.16 | ±0.07 |  |
|  |  | 0.08 | ±0.03 |  |
|  | Ca^2+^ | 1.54 | ±1.39 | 1.01 ± 0.26 |
|  |  | 0.71 | ±0.45 |  |
|  |  | 0.79 | ±0.71 |  |
|  | Co^2+^ | 1.13 | ±0.33 | 0.68 ± 0.30 |
|  |  | 0.11 | ±0.75 |  |
|  |  | 1.82 | ±1.82 |  |
| **MRS2-D216A/D220A** | Mg^2+^ | 0.47 | ±0.36 | 0.98 ± 0.25 |
|  |  | 1.33 | ±0.36 |  |
|  |  | 1.12 | ±0.40 |  |
|  | Ca^2+^ | 1.39 | ±1.27 | 0.74 ± 0.49 |
|  |  | 0.67 | ±0.32 |  |
|  |  | 0.81 | ±0.59 |  |
|  | Co^2+^ | 0.99 | ±0.26 | 1.37 ± 0.51 |
|  |  | 2.40 | ±1.03 |  |
|  |  | 0.73 | ±0.21 |  |

^a^ Data are extracted from fits to a one-site binding model that accounts for protein concentration.

^b^ Errors (±) are fitted errors from the one-site binding model fit.

^c^ Errors (±) are SEM from the n=3 K_d_ values reported, determined using three protein preparations.
